# A co-formulation of interferons alpha2b and gamma distinctively targets cell cycle in the glioblastoma-derived cell line U-87MG

**DOI:** 10.1186/s12885-023-11330-2

**Published:** 2023-08-29

**Authors:** Jamilet Miranda, Dania Vázquez-Blomquist, Ricardo Bringas, Jorge Fernandez-de-Cossio, Daniel Palenzuela, Lidia I. Novoa, Iraldo Bello-Rivero

**Affiliations:** 1 Bioinformatics Group, Center for Genetic Engineering and Biotechnology (CIGB), Havana, Cuba; 2 Pharmacogenomics Group, Center for Genetic Engineering and Biotechnology (CIGB), Havana, Cuba; 3 Clinical Assays Division, Center for Genetic Engineering and Biotechnology (CIGB), Havana, Cuba

**Keywords:** HeberFERON, Alpha interferon, Gamma interferon, Drug combination, U-87MG, Glioblastoma, Mitotic cell cycle, FOXM1, PLK1, AURKB, BIRC5(Survivin), CDC20, p53, STAT1

## Abstract

**Background:**

HeberFERON is a co-formulation of α2b and γ interferons, based on their synergism, which has shown its clinical superiority over individual interferons in basal cell carcinomas. In glioblastoma (GBM), HeberFERON has displayed promising preclinical and clinical results. This led us to design a microarray experiment aimed at identifying the molecular mechanisms involved in the distinctive effect of HeberFERON compared to the individual interferons in U-87MG model.

**Methods:**

Transcriptional expression profiling including a control (untreated) and three groups receiving α2b-interferon, γ-interferon and HeberFERON was performed using an Illumina HT-12 microarray platform. Unsupervised methods for gene and sample grouping, identification of differentially expressed genes, functional enrichment and network analysis computational biology methods were applied to identify distinctive transcription patterns of HeberFERON. Validation of most representative genes was performed by qPCR. For the cell cycle analysis of cells treated with HeberFERON for 24 h, 48 and 72 h we used flow cytometry.

**Results:**

The three treatments show different behavior based on the gene expression profiles. The enrichment analysis identified several mitotic cell cycle related events, in particular from prometaphase to anaphase, which are exclusively targeted by HeberFERON. The FOXM1 transcription factor network that is involved in several cell cycle phases and is highly expressed in GBMs, is significantly down regulated. Flow cytometry experiments corroborated the action of HeberFERON on the cell cycle in a dose and time dependent manner with a clear cellular arrest as of 24 h post-treatment. Despite the fact that p53 was not down-regulated, several genes involved in its regulatory activity were functionally enriched. Network analysis also revealed a strong relationship of p53 with genes targeted by HeberFERON. We propose a mechanistic model to explain this distinctive action, based on the simultaneous activation of PKR and ATF3, p53 phosphorylation changes, as well as its reduced MDM2 mediated ubiquitination and export from the nucleus to the cytoplasm. PLK1, AURKB, BIRC5 and CCNB1 genes, all regulated by FOXM1, also play central roles in this model. These and other interactions could explain a G2/M arrest and the effect of HeberFERON on the proliferation of U-87MG.

**Conclusions:**

We proposed molecular mechanisms underlying the distinctive behavior of HeberFERON compared to the treatments with the individual interferons in U-87MG model, where cell cycle related events were highly relevant.

**Supplementary Information:**

The online version contains supplementary material available at 10.1186/s12885-023-11330-2.

## Introduction

The molecular signaling networks that are underlying complex diseases limit the efficacy of single-drug treatments. Drug combinations targeting multiple elements of these networks have shown advantages over one-target therapies [1]. One example is the synergistic effect of the combination of type I and II interferons (IFNs) or their combinations with other cytokines [2–5]. On the other hand, the rapid introduction of genome-wide technologies and network biology approaches has contributed more in-depth studies of drug combination mechanisms.

The co-formulation of IFNs alpha2b and gamma as HeberFERON, is produced at the Center for Genetic Engineering and Biotechnology (CIGB). This product, based on the synergism between both types of IFNs, was registered for basal cell carcinomas where was demonstrated the clinical superiority of HeberFERON (IFN α/γ) over individual IFN treatments [6]. Additionally, HeberFERON was used off-label for other types of cancer with promising results [7]. Glioblastoma (GBM) is a type of cancer where HeberFERON has shown clinically encouraging results [8].

IFNs as cytokines display pleiotropic actions including antiviral and growth-inhibitory effects through several intracellular pathways from the type I and II receptors [9]. Signaling crosstalk between IFN-α/β and -γ induce stronger activation of STAT transcription factors [10].

The aim of this paper is to help unravel the mechanism explaining the distinctive behavior of the HeberFERON combination over the individual IFN treatments through a transcriptomic profile study in the human glioblastoma derived cell line U-87MG. The integrative analysis of microarray data allowed us to propose a model to explain the biological outcomes and we validated cell cycle as one of the most important processes involved. This report is the first high throughput experiment to investigate the distinctive effect of HeberFERON in the context of GBM.

## Materials and methods

### Reagents

The recombinant IFNs, rIFN-α2b and rIFN-γ, are produced at CIGB, Havana, Cuba. A pharmaceutically stable formulation that combines both IFN-α2b and IFN-γ (HeberFERON) is also manufactured at CIGB [6, 7].

### Cell cultures

The human glioma cell line U-87MG (ECACC Product number 89,081,402, Salisbury, Wiltshire, UK) (TP53wt, PTENmut) was maintained in the complete MEM medium (Sigma, USA), supplemented with 10% Fetal Bovine Serum, 2mM glutamine and 50 µg/ mL of gentamicin (All Gibco) in a humidified atmosphere of 5% CO_2_ at 37 °C. Cells were grown at a cellular density of 35 000 cells/ cm^2^ in 75 cm^2^ flasks. After 24 h, cells were treated with HeberFERON at IC50 or with equivalent amounts of rIFN-α2b and rIFN-γ using the same culture medium, and incubated for 72 h. Untreated cells were included in the experimental setup.

### Cell viability assay and counting

An MTT assay (3-(4,5-dimethylthiazol-2-yl)-2,5-diphenyltetrazolium bromide) was used for U-87MG viability studies. Briefly, cells were placed in 96-well culture plates (10^4^ cells/ well). After 24 h, cells were treated with 1:2 serial dilutions from 78 to 5000 total IU/mL of HeberFERON in triplicates for 72 h. At the end of the treatment, 20 µL of MTT (5 mg/mL) was added to each well. The cells were incubated for another 4 h in a humidified atmosphere of 5% CO_2_ at 37 °C, and 100 µL of 50% isobutyl alcohol-10% SDS solution was added to each well. Absorbance was measured at 540 nm and we calculated the growth inhibition ratio. Three separate experiments were performed. The half-inhibitory concentration values (IC50) were obtained from the MTT viability curves using Calcusyn software (version 2.1, Biosoft 1996–2007).

Cells were counted in a hemocytometer diluted with 0.4% Trypan blue solution. Duplicate 175cm^2^ flasks seeded with U-87MG cells (35 000 cells per cm^2^) were treated for each condition and we counted the cells after 72 h. The number of cells is given as the average ± SD (standard deviation) in absolute number of cells or in relation to the untreated control that was considered 100%. For kinetic treatment with HeberFERON, duplicate 25cm^2^ flasks seeded with U-87MG cells (30 000 cells per cm^2^) were treated with IC50 dose of HeberFERON and we counted the cells after 24 h, 48 and 72 h.

### Experimental design

The experimental design consisted of four groups or conditions with six biological replicates each, including cells, i.e. treated with IFNα2b, treated with IFNγ, treated with HeberFERON and an untreated control group.

### RNA purification

After 72 h of incubation with the IFNs, the medium was discarded and cells were washed once with phosphate saline buffer. Cells were scraped off in buffer RLT with 143mM β-mercaptoethanol and total RNA purification proceeded following the instructions of RNeasy Plus minikit (Qiagen, USA). For quality control of total RNA we used spectrophotometric readings of optic density (OD) at 260 and 280 nm in Nanodrop 1000 (ThermoFisher, USA) to determine concentration (> 80ng/µL) and OD260/280 ratio (1.8–2.2). Additionally, RIN (7–10) was calculated by capillary electrophoresis in a Bioanalyzer (Agilent, Waldbronn, Germany). We sent 2.5 µg (100 ng/µL) of each total RNA sample to McGill University and Génome Québec Innovation Centre (Montréal, Québec, Canada) for the microarray experiment in Illumina HumanHT-12 v4 Expression BeadChip platform.

### Basic microarray data analysis provided by the Génome Québec Innovation Centre

The microarray experiment and a basic bioinformatics analysis were performed as a custom service at McGill University and Génome Québec Innovation Centre (Montréal, Canada). This service included quality control, preprocessing, exploratory and differential expression analysis. As a result of the quality control, none of the arrays was removed. Preprocessing included imputation for missing value using kNN algorithm, background correction and normalization using the neqc methodology described by W Shi, A Oshlack and GK Smyth [11]. For differential expression analysis the Bioconductor Limma package [12] was used. Statistical tests contrasting different treatments were performed (Moderated t-tests) [13, 14]. A linear model was fit to each gene using treatment as variables and assumed random sampling (no pairing). The Benjamini-Hochberg was used for false-discovery rate (FDR) estimation [15].

### Additional Bioinformatics Analysis

Two-dimensional hierarchical clustering analysis of log2 standardized expression profiles based on Spearman’s rank correlation and average linkage was performed using TIGR MeV analysis software (The Institute for Genomic Research, USA) [16, 17].

To select gene expression changes that distinguish the HeberFERON treatment, we compared transcription levels of treated groups against control samples. Genes with a fold change greater than 2 (|log_2_FC| >=1) and adjusted p values of less than 0.05 (Adj p < 0.05) were considered “Differentially Expressed Genes” (DEGs) and used for later bioinformatics analyses. We used Venn diagrams and a scatter volcano plot that provided a summary of test statistics for DEGs.

For the gene list functional enrichment analysis, the bioinformatics tools ToppGene suite (ToppFun) [18], GeneCodis [19, 20], David [21, 22] and BioPlanet resource were used [23]. The enrichment analysis was carried out against the following data sources: Gene Ontology (GO) [24] and biological pathways in KEGG [25, 26], REACTOME [27, 28] and Pathway Interaction Database (PID) [29]. A cutoff value of the adjusted p value < = 0.05 was set for considering an event to be significant.

Additionally, we used Gene Set Enrichment Analysis (GSEA, version 4.1.0 for windows) [30]. Gene lists ordered by fold change were provided as input. The pre-ranked gene list option was used for REACTOME pathway database sets. We downloaded MSigdb v7.2 gmt files from: http://www.gsea-msigdb.org/gsea/downloads.jsp. The permutation-based p-value was corrected for multiple testing to produce a FDR q-value that ranges from 0 (highly significant) to 1 (non-significant). The criteria used for statistical significance was a Nominal p-value threshold of 0.05 and a FDR of 0.25, as recommended by the GSEA software.

BisoGenet Cytoscape plugin [31], available from Cytoscape Application Manager, was used to generate protein-protein interaction (PPI) networks. Venn Diagrams were generated using the web application at: https://bioinformatics.psb.ugent.be/webtools/Venn/. For data visualization and analyses to explore brain tumor expression datasets, we used the GlioVis Web Application [32]. We selected as input the GBMLGG RNA-seq dataset that contain GBM (n = 152) and Low Grade Glioma (n = 515) samples. Tukey’s Honest Significant Difference (HSD) test was used to make all pairwise comparisons between brain tumor subtypes. For the pairwise statistical analysis between treatments an ANOVA with a Tukey’s multiple comparisons test was performed using Prism GraphPad Version 6.0 Software. Additional statistical analyses were performed in Bioconductor in R language (http://www.r-project.org/).

### Real-time PCR-based gene expression validation

Complementary (c) DNAs were obtained from 500 ng of total RNAs, using the Invitrogen SuperScript™ III First-Strand Synthesis SuperMix kit for qRT-PCR (Invitrogen, Carlsbang, USA) following the manufacturer’s instructions. The qPCR reactions were set up in 20 µL with 300 nM of oligonucleotides (List in Table S1 in Additional data file 1), 10 times diluted cDNAs and ABsolute QPCR SYBR Green Mixes (Thermo scientific, ABgene, UK) using three replicates per sample. The runs were carried out in an RT-Cycler (CapitalBio, China) using the standard controls and program [33]. REST 2009 [34] was used to report a Change Factor in gene levels after the treatment for 72 h with IFNα2b, IFNγ and HeberFERON in relation to untreated cells after the normalization with GAPDH and HMBS as reference genes. Increases and decreases of gene levels are reported as UP and DOWN, respectively, with associated p values [35].

### Cell cycle analysis

The U87-MG [36] cells were seeded in 6-well plates at 30 000 cells per cm^2^ and treated with HeberFERON and IFNα2b or IFNγ at the HeberFERON IC50 (4000 IU/mL) dose and the equivalents for individual IFNs after 24 h. Additional assays using 0.25XIC50 (1000 IU/mL) and 2.5 × (10,000 IU/mL) of HeberFERON were also carried out. Adherent cells were collected by trypsinization and washed twice with PBS after 24 h, 48 and 72 h. Methanol (with a final concentration of 78%) was added to the cells, drop by drop while gently shaking, and further washed twice. Cells were then stained with 20 µg/mL of Propidium Iodide (PI) and 100 µg/mL of RNase A for 30 min at 37º in the dark. Finally the stained cells were analyzed and studied by flow cytometry at 488 nm (Partec CyFlow® space, GmbH, Germany; equipped with FloMax 2.9 software). Detection graphs at FL2 channel vs. counts were generated.

## Results

### Effects of HeberFERON on U-87MG proliferation

A viability anti-proliferative study of HeberFERON on the U-87MG cell line using the MTT assay revealed a dose-dependent effect with an IC50 value of 4000 IU/mL (Fig. S1A in Additional data file 2). Moreover, HeberFERON reduced U-87MG cell counts in 50% using the IC50 dose. Cell count reduction was not observed with individual IFNα2b or IFNγ at equivalent doses as in HeberFERON (Fig. S1B in Additional data file 2). Also the kinetics experiment of HeberFERON treatment revealed a reduction of cell count at 72 h of treatment and not before (Fig. S1C in Additional data file 2).

### Unsupervised methods revealed the four sample groups

**Fig. 1:**
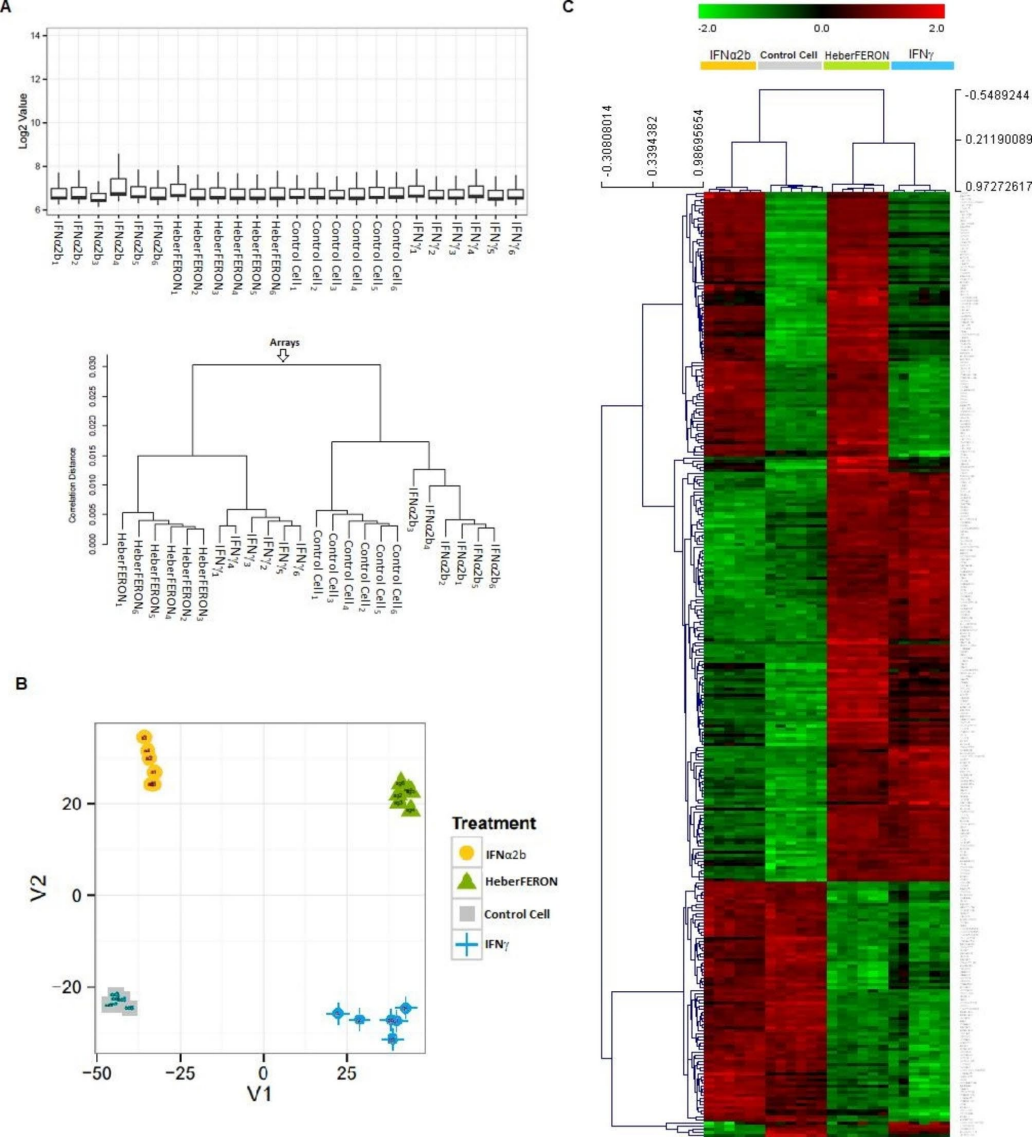
**Diagnostic plots and unsupervised clustering analyses of gene expression profiles**. (**A**) Boxplot visualization of log2 expression values and one-dimensional hierarchical clustering of all sample groups: control cell samples and those treated with IFNα2b, IFNγ and HeberFERON. (**B**) Multidimensional Scaling (MDS) of flat pattern filtered data. (**C**) Two dimensional hierarchical clustering of genes expression profiles with the highest SD between samples. On top, samples are grouped based on the similarity of their expression profiles

The boxplot of microarray gene expression values shows a similar picture for all samples and one-dimensional clustering separates the samples into the four experimental groups (Fig. 1A). Additionally, 2D multidimensional scaling shows the samples for each condition closely grouped around each of the four corners while samples from different conditions are distantly located (Fig. 1B). Bidimensional hierarchical clustering (Fig. 1C) was applied to genes showing the most variable expressions (SD > 1). In all cases, the charts show that the four experimental groups are perfectly separated. In addition, clustering shows four well distinguishable sets of genes (vertical axis) with different behavior in each one of the four conditions.

### Differentially expressed genes by treatments with interferons

**Fig. 2:**
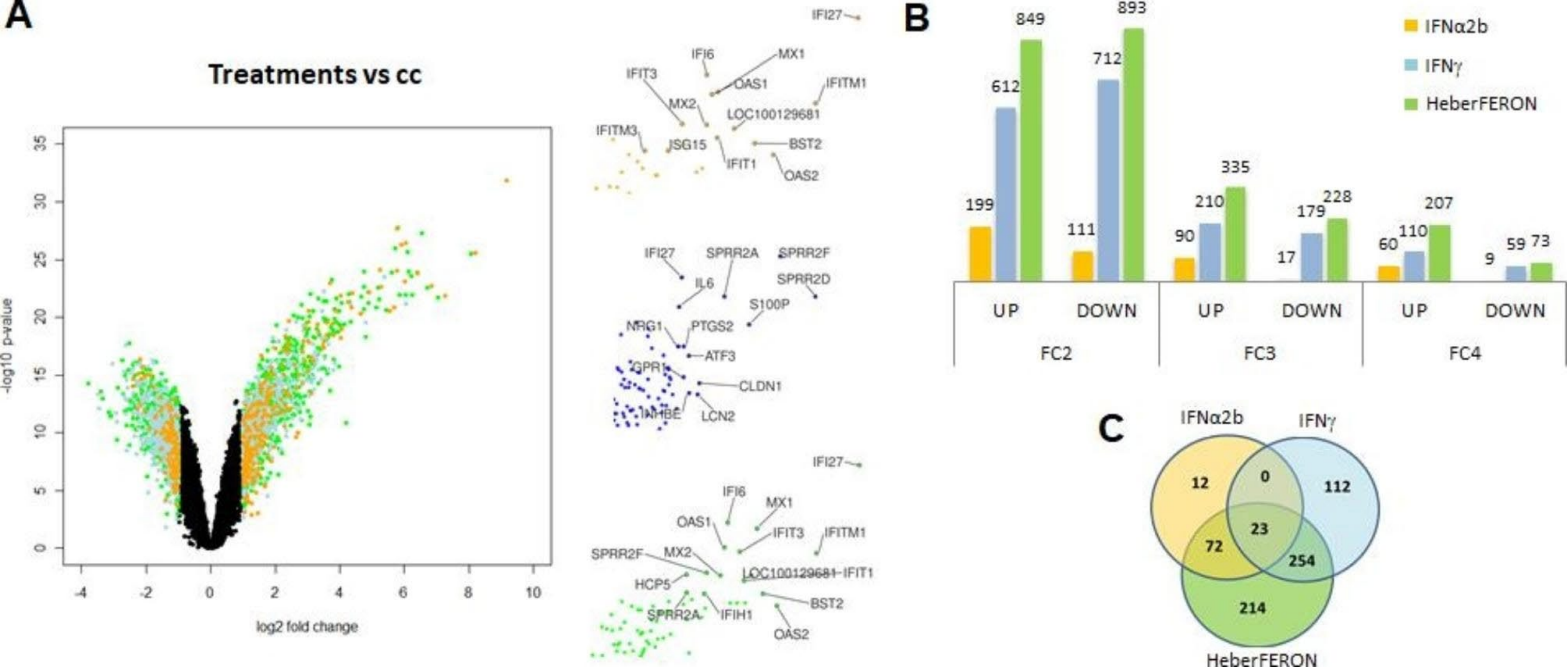
**Analysis of differentially expressed genes by the action of IFN treatments**. (**A**) Volcano plot visualizes significant changes between the control group and the treatment with HeberFERON (green dots), with IFNα2b (orange dots) and with IFNγ (light blue dots). On the right three zoomed plot of differentially over-expressed genes for each treatment, top genes are labeled. (**B**) The number of DEGs for different fold changes and signs of expression. (**C**) The Venn diagram of sets of DEGs is represented for the three treatment groups (|FC|>=3): IFNα2b (orange circle), IFNγ (light blue circle) and HeberFERON (green circle). Numbers refer to DEGs that are specific to one of the treatments or common to two or to the three treatments. Differentially expressed genes were considered those with an absolute log_2_FC higher than 1 and adjusted p < 0.05, they are represented in the plot by non-black dots

The volcano plot shows the p-values vs. FC values for each gene and treatment in the study (Fig. 2A) where orange, light blue and green dots represent values for IFNα2b, IFNγ and HeberFERON treatments, respectively. DEGs are located to the left (down-regulated genes) and to the right (up-regulated genes). The genes with the highest significant changes were predominantly from the HeberFERON treatment group. It is of note that most of the DEGs by the HeberFERON treatment that were down-regulated by a factor of more than 4, were not differentially expressed by the individual IFN treatments. The numbers of up- and down-regulated genes for three different fold-change cut-off values (2, 3, 4) are plotted in Fig. 2B. The smallest number of DEGs is in the IFNα2b treatment, while HeberFERON produces the largest number of DEGs. The Venn diagram in Fig. 2C was built with sets of genes with a fold change of more than three (|FC|>3), in each of the three treatment groups. It shows 214 genes, out of 563, differentially expressed by HeberFERON, which are specific for this treatment.

### HeberFERON shares some DEGs with IFNα2b and IFNγ

The enrichment analysis of GO terms was conducted for 23 genes that are common to the three treatments (Fig. 2C), using David. In Table S2 in Additional data file 1 we summarize the most significant biological processes (adjusted p value < 0.05). The most significant terms shared by the three treatments were “the defense response to virus” and “the innate immune response”. Afterwards, other events (e.g. “antigen processing and presentation of endogenous peptide antigen via MHC class I”, “positive regulation of T cell mediated cytotoxicity”, and “type I IFN signaling pathway”) were also included. All these are known to be activated by the action of IFNs.

### Cell cycle regulation by HeberFERON

**Fig. 3:**
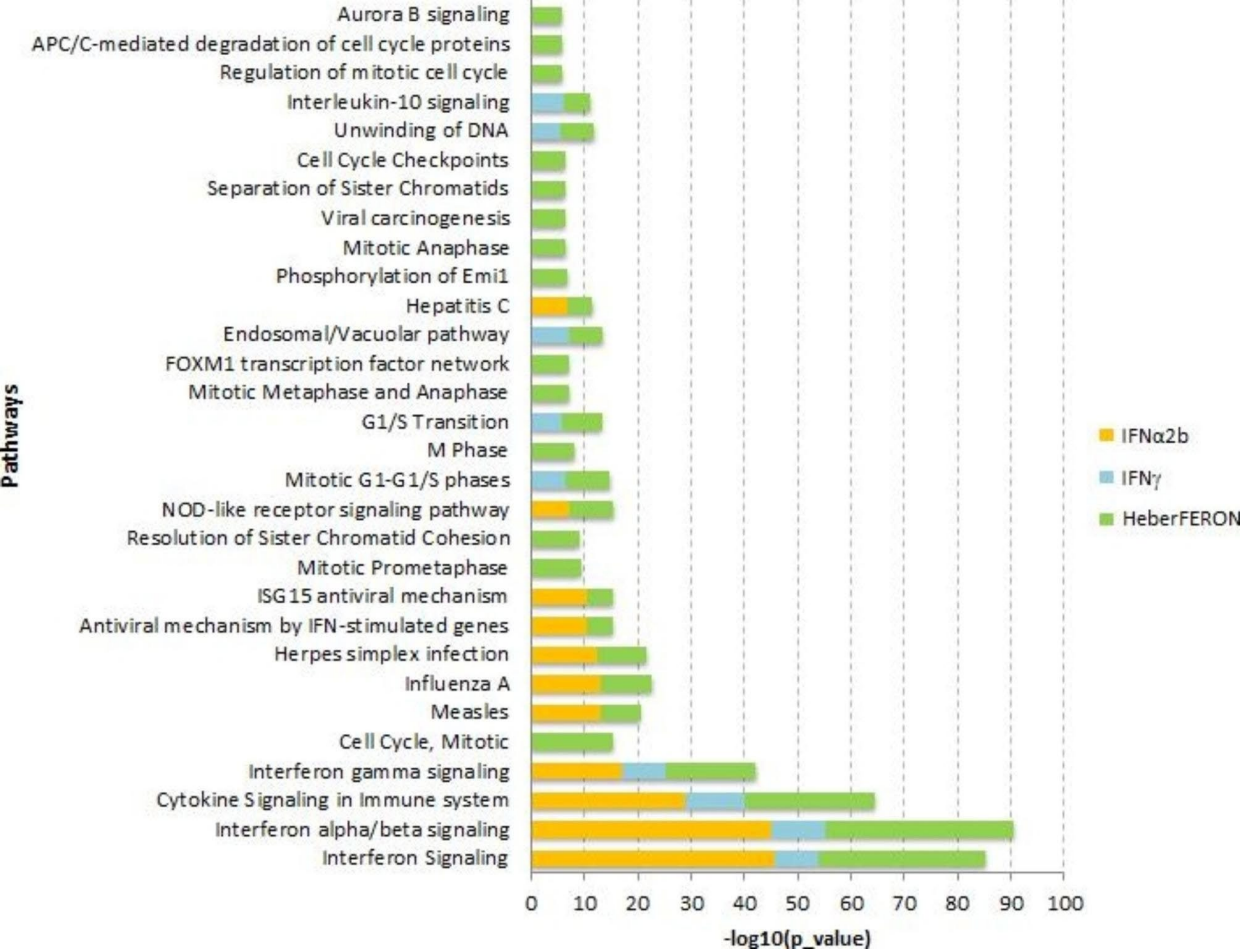
**Results of ToppGene enrichment analysis for treatments**. DEGs with Fold-Change three or higher were subjected to the enrichment analysis. Top 30 Signaling Pathways were sorted by significance from bottom to top. Orange, blue and green bars correspond to IFNα2b, IFNγ and HeberFERON, respectively. The X axis represents the level of significance (-log_10_(p-value)) of the enrichment scores

Next we performed an enrichment analysis of sets of genes, using ToppGene, with |FC|>3 for each treatment. The results for each treatment were mixed and the terms were reordered according to the highest level of significance (p-value). The stacked bar chart in Fig. 3 shows the top 30 signaling pathways according to their significance (lowest p-value). The –log_10_ (p-value) for each treatment is plotted in a stacked horizontal bar. As expected, the most significant pathways are those related to cytokine and IFN signaling.

The first four pathways are enriched by the three treatments. Most of the 23 DEGs common to the three treatments (Fig. 2C) are involved in these four pathways. The next most significant pathway was the “Mitotic Cell Cycle”. The latter, together with other pathways related to cell cycle such as “Mitotic Prometaphase”, “Resolution of Sister Chromatid Cohesion”, “Mitotic Metaphase and Anaphase”, “FOXM1 transcription factor network”, “Cell Cycle Checkpoints”, “Aurora B signaling”, “Phosphorylation of Emi1” and “APC/C-mediated degradation of cell cycle proteins”, were exclusively observed in the HeberFERON treatment. In the case of “G1/S Transition” and “Mitotic G1-G1/S phases” pathways, IFNγ and HeberFERON treatments show similar behavior, suggesting that the IFNγ alone may also induce a certain inhibition of these cell cycle events.

Additionally, a comparative pathway enrichment analysis (CPEA) between HeberFERON and individual IFN treatments was conceived as follows: (1) set of DEGs by HeberFERON treatment with |FC|>3 were selected; (2) for individual IFN treatments, a less restrictive cutoff (|FC|>=2) was used; (3) the three sets of DEGs were subjected to the enrichment analysis; (4) from the list of pathways enriched by HeberFERON, those enriched by either one of the individual IFNs were excluded. The resulting list of pathways would be considered as distinctively activated by HeberFERON.

**Table 1:** **List of the top BioPlanet significant pathways regulated by HeberFERON as the result of CPEA.**

Pathway_id	Description	Adj p value	Genes involved
bioplanet_1345	FOXM1 transcription factor network	1.87E-05	AURKB, BIRC5, CCNA2, CENPF, FOXM1, CENPA, CCNB2, CCNB1, PLK1, NEK2
bioplanet_592	Aurora B signaling	1.03E-04	STMN1, AURKB, CDCA8, BIRC5, CENPA, NCAPG, INCENP, KIF23, KIF20A
bioplanet_1622	Phosphorylation of Emi1	3.82E-04	FBXO5, CDC20, CCNB1, PLK1
bioplanet_67	Stathmin and breast cancer resistance to antimicrotubule agents	1.40E-03	HIST1H4C, STMN1, CREB1, OAS1, USP18, HLA-B, STAT1, GBP1, CXCL10, PRKACB, IRF1, IFIT2, KAT2B, IFI6, TAP1, ISG15, EIF2AK2, RNF14, CCNB1, STAT2, PSMB9, IRF9
bioplanet_1327	Polo-like kinase 1 (PLK1) pathway	1.79E-03	FBXO5, CDC20, PRC1, INCENP, CCNB1, SPC24, PLK1, KIF20A
bioplanet_1757	Hypertrophy pathway	5.99E-03	IFRD1, ATF3, VEGFA, CYR61, IL1A
bioplanet_1391	APC/C activator regulation between G1/S and early anaphase	7.36E-03	FBXO5, CDC20, UBE2C, CCNB1, PLK1
bioplanet_1363	Delta Np63 pathway	1.09E-02	NRG1, FASN, RRAD, TOP2A, CCNB2, IL1A, RAB38
bioplanet_235	Cytokine-cytokine receptor interaction	1.31E-02	TNFSF13B, CSF3, IL11RA, INHBE, CXCL5, TNFSF10, CSF1R, CXCL10, CCL2, IL20RB, CCL20, CXCL2, LEP, VEGFA, IL1A, IL6, IL24, TNFRSF10D, CCL8
bioplanet_884	APC/C- and Cdc20-mediated degradation of Nek2A	1.47E-02	MAD2L1, CDC20, UBE2C, CCNB1, NEK2
bioplanet_1511	Cyclin A/B1-associated events during G2/M transition	1.49E-02	CCNA2, CCNB2, CCNB1, PLK1
bioplanet_195	p53 activity regulation	1.92E-02	GADD45A, KAT2B, SESN2, CCNA2, CCNE2, SHISA5, CCNB2, PMAIP1, GTSE1, CCNB1, CCND2
bioplanet_1575	Kinesins	1.95E-02	KIF22, KIFC1, KIF11, KIF23, KIF20A
bioplanet_1139	MicroRNA regulation of DNA damage response	2.19E-02	CREB1, MCM7, GADD45A, CCNE2, CCNB2, PMAIP1, CCNB1, CCND2
bioplanet_1409	Mitotic prometaphase	2.82E-02	ZWINT, CDC20, CENPF, CENPA, INCENP, PLK1

**Fig. 4:**
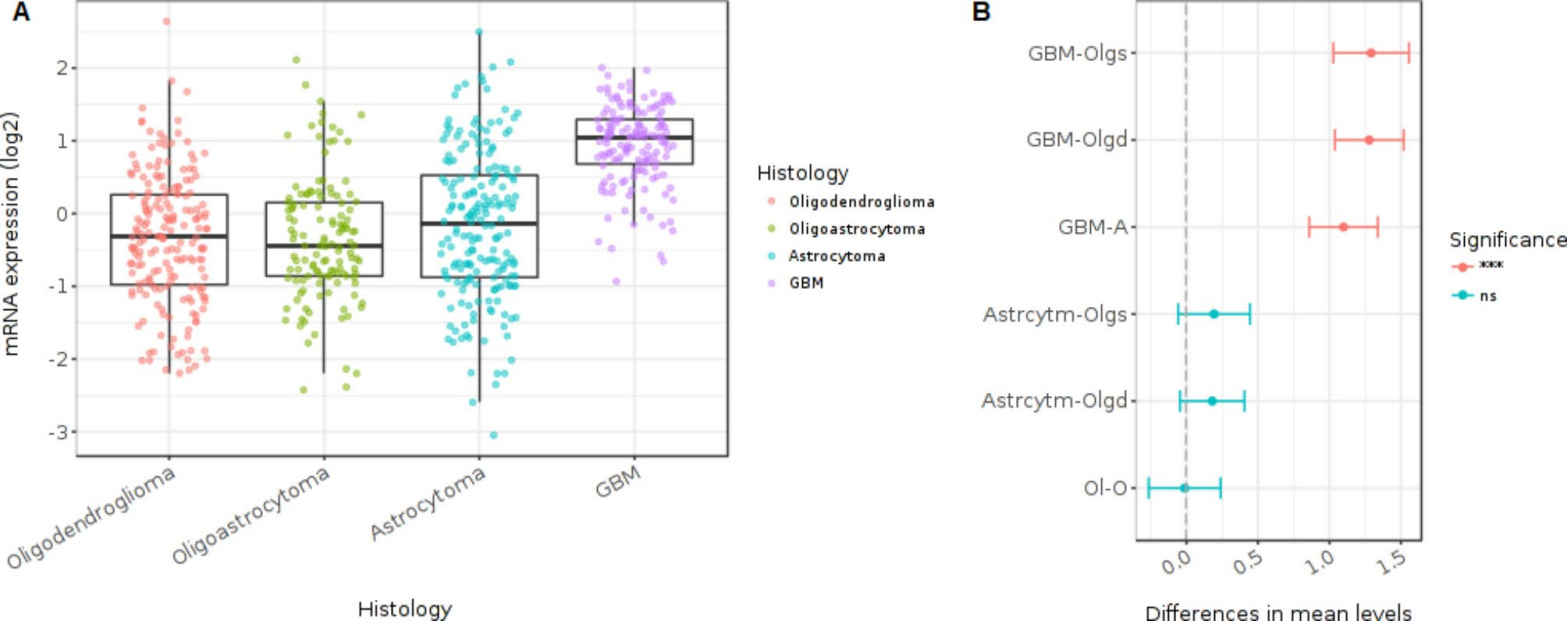
**FOXM1 expression in brain tumors**. The figure was generated with GlioVis application including data from 515 samples of low grade gliomas (oligodendrogliomas, oligoastrocytomas and astrocytomas) and 152 samples of GMB from TCGA GMBLGG dataset. (**A**) FOXM1 mRNA expression levels of the four types of brain tumors. (**B**) Tukey’s Honest Significant Difference results of pairwise comparisons between types of brain tumors. The plot shows the difference between pairs, the 95% confidence interval and use to asterisks according the level of significance of differences (p-value of the pairwise comparisons), ***p < 0.001; ns, not significant

Table 1 shows a list of these pathways ordered by an ascending adjusted p-value. Out of the 15 pathways listed, the most significant event was the “FOXM1-transcription factor network”. The genes responsible for this enrichment are listed in Table 1 (AURKB, BIRC5, CCNB1, CCNB2, CCNA2, CENPA, CENPF, NEK2 and PLK1). FOXM1 is highly expressed in GBM (Fig. 4A), its expression is significantly higher in GBM than in other brain tumors (Fig. 4B). Consequently genes regulated by FOXM1 have a similar behavior (Fig. S2 in Additional data file 2). Together with mitotic cell cycle related pathways already found by the ToppGene enrichment analysis, we observed “p53 activity regulation” as a new enriched event. Although p53 was not differentially expressed by HeberFERON, several genes participating in its signaling including cyclins (CCNA2, CCNE2, CCNB1, CCNB2, CCND2) were down-regulated by HeberFERON.

**Fig. 5:**
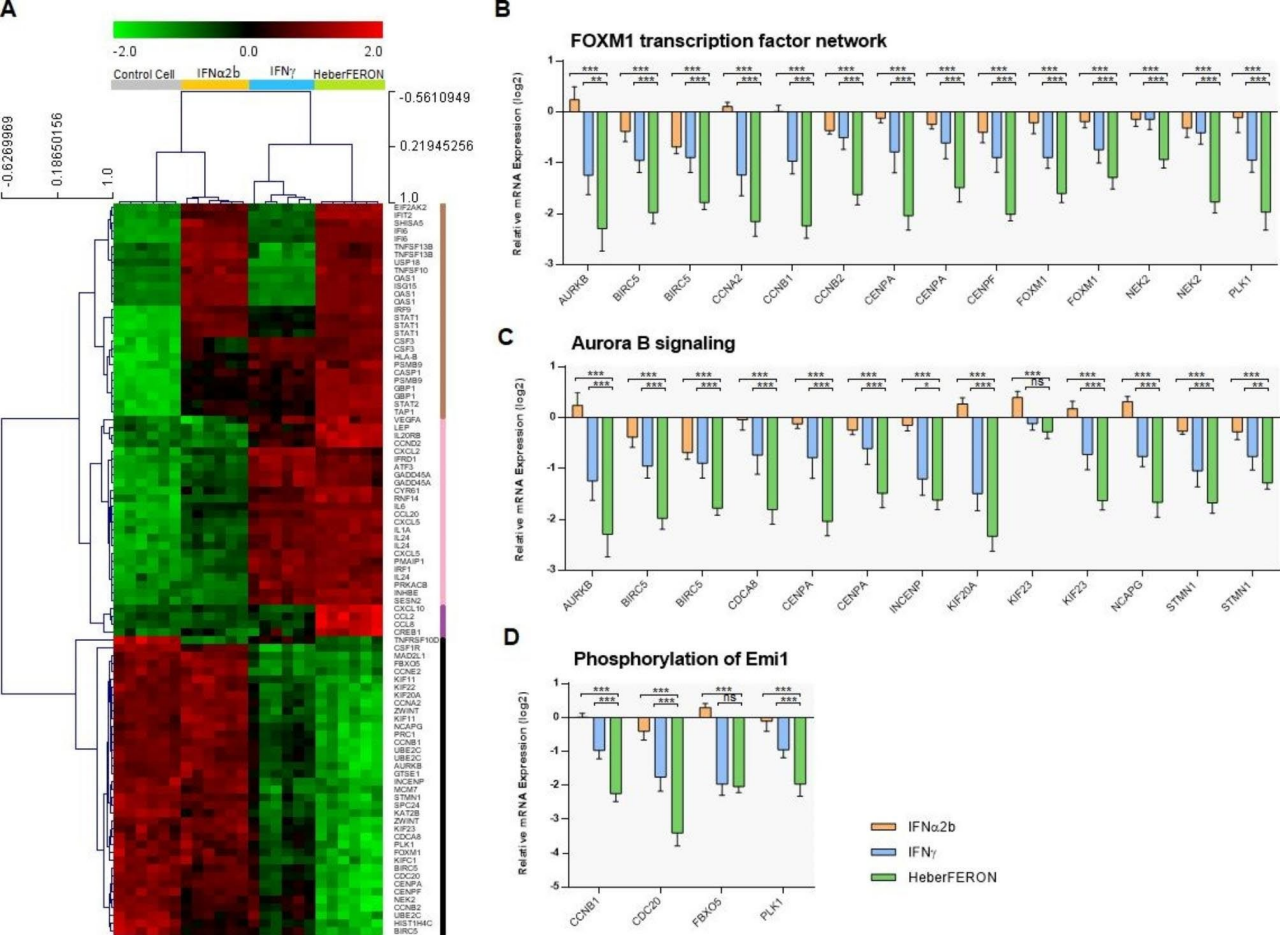
**Gene expression analysis of genes resulting from the CPEA of the BioPlanet pathways**. (**A**) The bidimensional hierarchical clustering of all genes resulting from the CPEA in control cell samples and those treated with IFNα2b, IFNγ and HeberFERON. The four colors on the vertical line to the right of the heatmap identify four different gene clusters: first cluster (brown) groups genes up-regulated by IFNα2b and HeberFERON; second cluster (light pink) groups genes up-regulated by IFNγ and HeberFERON; third cluster (magenta) groups a small set of genes up-regulated by HeberFERON; and the fourth cluster (black) groups genes down-regulated by the HeberFERON treatment. (**B**), (**C**) and (**D**) log_2_ expression relative to averaged controls of genes in the top three enriched pathways resulting from the CPEA: “FOXM1 transcription factor network”, “Aurora B signaling” and “Phosphorylation of Emi1”, respectively. Differential expression analysis was conducted with Limma package [12]. Statistical tests contrasting different treatments were performed (Moderated t-tests). Statistical significance is represented as *** Adj p < 0.001; ** Adj p < 0.01; * Adj p < 0.05; ns, not significant

From the 15 enriched pathways we identified 75 DEGs. The unsupervised bidimensional clustering in Fig. 5A groups the expression profiles in four main gene clusters (vertically) and the four samples (horizontally). The first two gene clusters (brown and pink lines) contain genes that are up-regulated by IFNα2b and IFNγ, respectively. HeberFERON up-regulates most genes in both clusters. The third cluster (magenta line) contains a few chemokines while the fourth cluster (black line) contains genes involved in several enriched cell cycle events, which are strongly down-regulated by HeberFERON. Figure 5B C and 5D show the expression profiles of genes related to the first three events listed in Table 1 (“FOXM1 transcription factor network”, “Aurora B signaling” and “Phosphorylation of Emi1”). These genes belong to the forth cluster in Fig. 5A.

Furthermore, we applied a functional enrichment analysis using GSEA, a method that does not require any filtering by a threshold. As input, we provided lists of all genes in the microarrays ranked by fold change. The method identifies enriched terms at the top or bottom of the ranked list. In the supplementary figure S3 in Additional data file 2, we show GSEA results for the effect of HeberFERON and IFNγ. Pathways are ranked by the normalized enrichment score (NES) of the HeberFERON treatment. The top 12 pathways on the list are related to cell cycle and are clearly more down-regulated by the HeberFERON treatment (Fig. S3A). Figures S3B and C show the enrichment plots and the most relevant genes from the core enrichment set (CES) of events over-represented only by HeberFERON: “Deposition of new CENPA containing nucleosomes at the centromere” and “Kinesins”. Additionally, in figure S4 in Additional data file 2 the expression levels of the genes involved in the cell cycle show that most of them are more down-regulated by HeberFERON than by the individual IFN treatments. These results reinforce cell cycle related events as distinctively targeted by HeberFERON compared to individual IFN treatments.

### Network analysis of genes targeted by HeberFERON

**Fig. 6:**
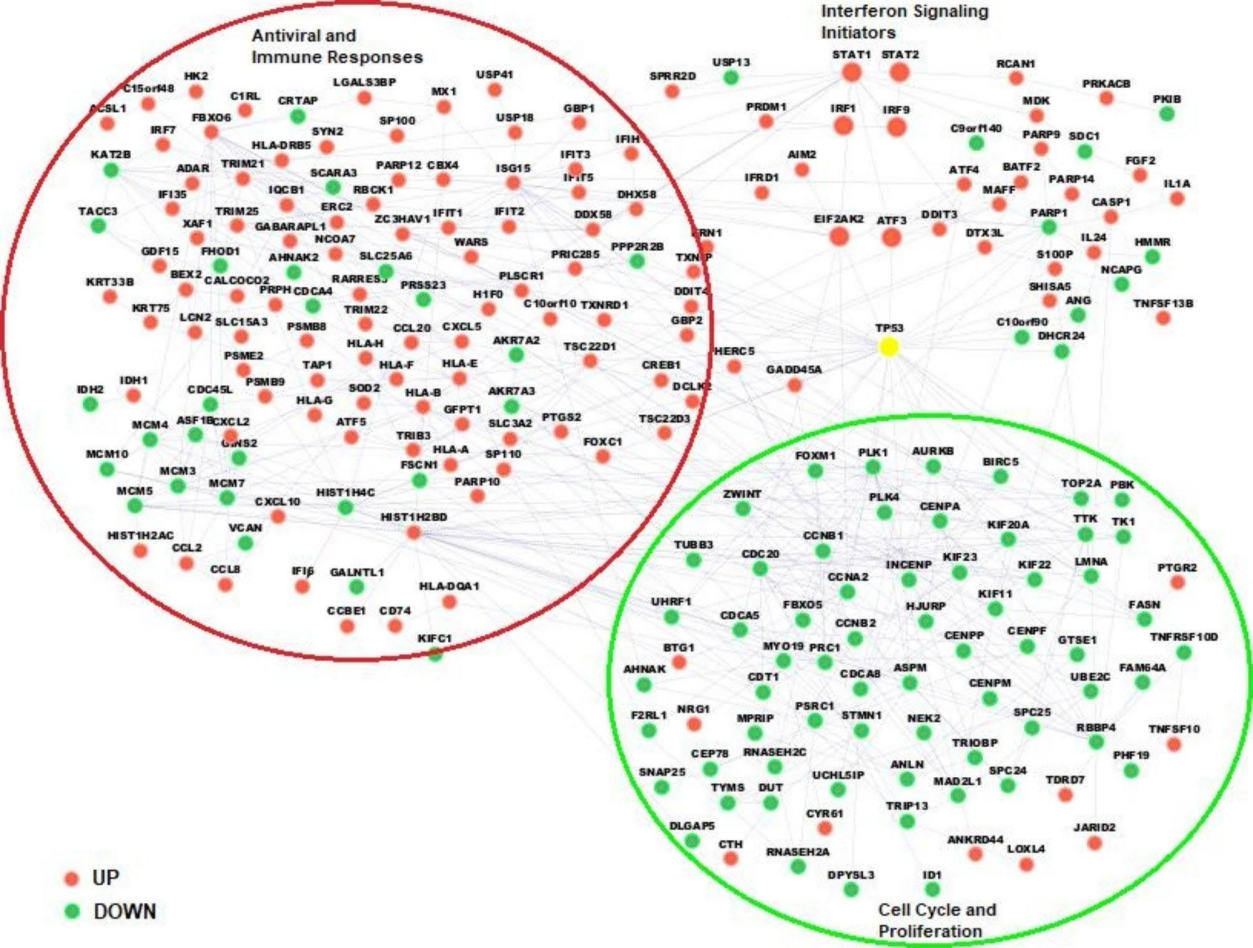
**Network of DEGs by HeberFERON treatment plus p53 gene**. A total of 230 nodes are represented. The rest of the 563 DEGs were not connected or were part of small sub-networks. DEGs in red and green represent up-regulated and down-regulated DEGs, respectively. In yellow, we show the node representing the p53 tumor suppressor, the one with the higher degree. The network was generated by the BisoGenet CytoScape application

Figure 6 shows a network of DEGs by HeberFERON plus p53 composed by 230 connected nodes. p53 was added to the network because its activity regulation was one of the CPEA enriched events. It is noteworthy that the node with the highest degree is that representing tumor suppressor p53, suggesting the role of this protein in mediating HeberFERON action. It is important to note that in U-87MG cell line the p53 is in its wildtype status.

This network shows the high interconnection between the DEGs from the STATs, but also subsets of genes participating in common pathways for IFN treatments as IFN signaling, antiviral and immune responses. The p53 encoding gene (TP53) is a hub node from where the signaling transduction cascade connects to biomarkers participating in cell cycle events (PLK1, AURKB, ZWINT, CCNA2), proliferation (TOP2A, TK1, PBK, TTK) or replication (MCM family members). Most of these biomarkers are down-regulated by HeberFERON.

**Fig. 7:**
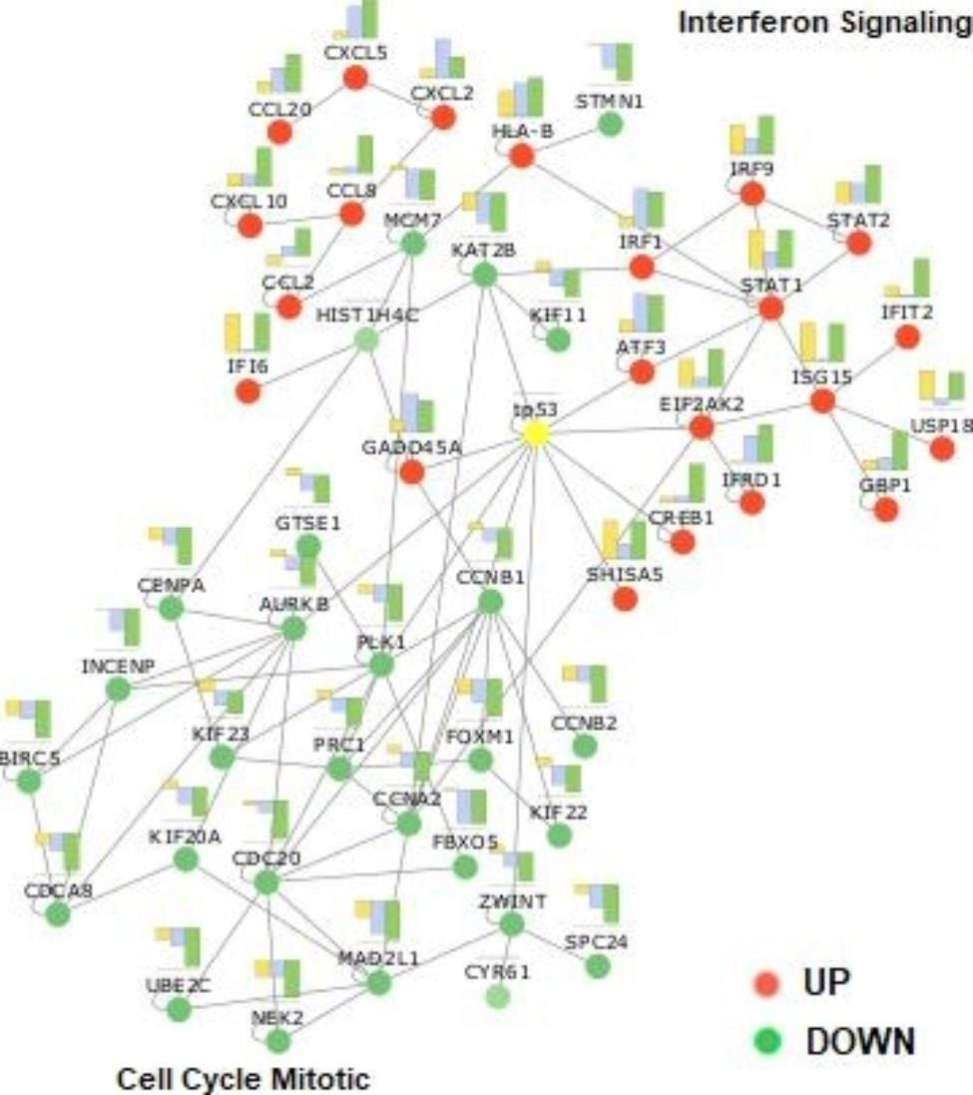
**Network of the interconnected genes (50 of 75) selected from the CPEA plus p53**. The red and green dots represent up-regulated and down-regulated DEGs, respectively. The yellow dot is the node representing the p53 tumor suppressor. For each node, a bar chart shows the expression level of each gene in the three experimental groups: orange, blue and green bars correspond to IFNα2b, IFNγ and HeberFERON, respectively. The network was generated by the BisoGenet CytoScape application

These effects are observed better when we built a network with only the 75 genes resulting from the CPEA plus p53 (Fig. 7). A 50 connected nodes network is composed of two main sub-networks. The first sub-network is composed of key mediators of IFN Signaling, including STAT1, STAT2 and IRFs genes, predominantly up-regulated by HeberFERON. A second sub-network included genes that are involved in cell cycle mitotic events predominantly down-regulated by HeberFERON. Here, it is evident that the p53 encoding gene (TP53) is located in the interface between both sub-networks.

A PubMed enrichment analysis using ToppFun identified a signature of poor prognosis in the Proneural subtype of GBM (PMID: 22,242,177). The genes involved in this signature are closely related to STAT1, which is up-regulated by HeberFERON. The proximity of the IFIT1, IFIT3, ISG15, MX1, STAT1 and USP18 signature genes in the interaction network is observed in Fig. 6. In supplementary figure S5 in Additional data file 2 we show the expression levels of genes belonging to this poor prognosis signature for each IFN treatment.

### Validation of regulated cell cycle gene expression by qPCR

**Fig. 8:**
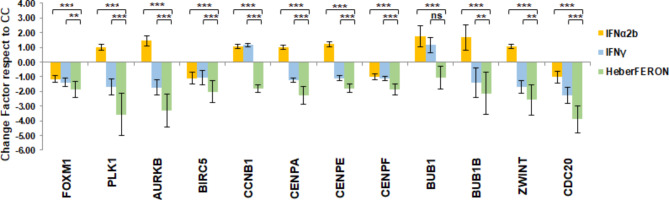
**Fold change of transcript levels for genes involved in cell cycle regulation determined by qPCR**. Fold change in transcript levels compared to the untreated controls are shown for the treatment with IFNα2b, IFNg or HeberFERON and the standard error associated with the measurements. The statistically significant differences according to REST2009 are represented as *** p < 0.001; ** p < 0.01; ns, not significant

We performed qPCR validation of a set of genes participating in cell cycle regulation, among them FOXM1 and members of its regulatory network and prometaphase proteins, PLK1, AURKB, BIRC5, CCNB1, CENPA, CENPF and ZWINT. We also included some other genes encoding proteins participating in the spindle checkpoint as CDC20, BUB1, BUB1R and CENPE. All of them showed a greater decrease in expression with the HeberFERON treatment (Fig. 8).

### Cell cycle analysis

**Fig. 9:**
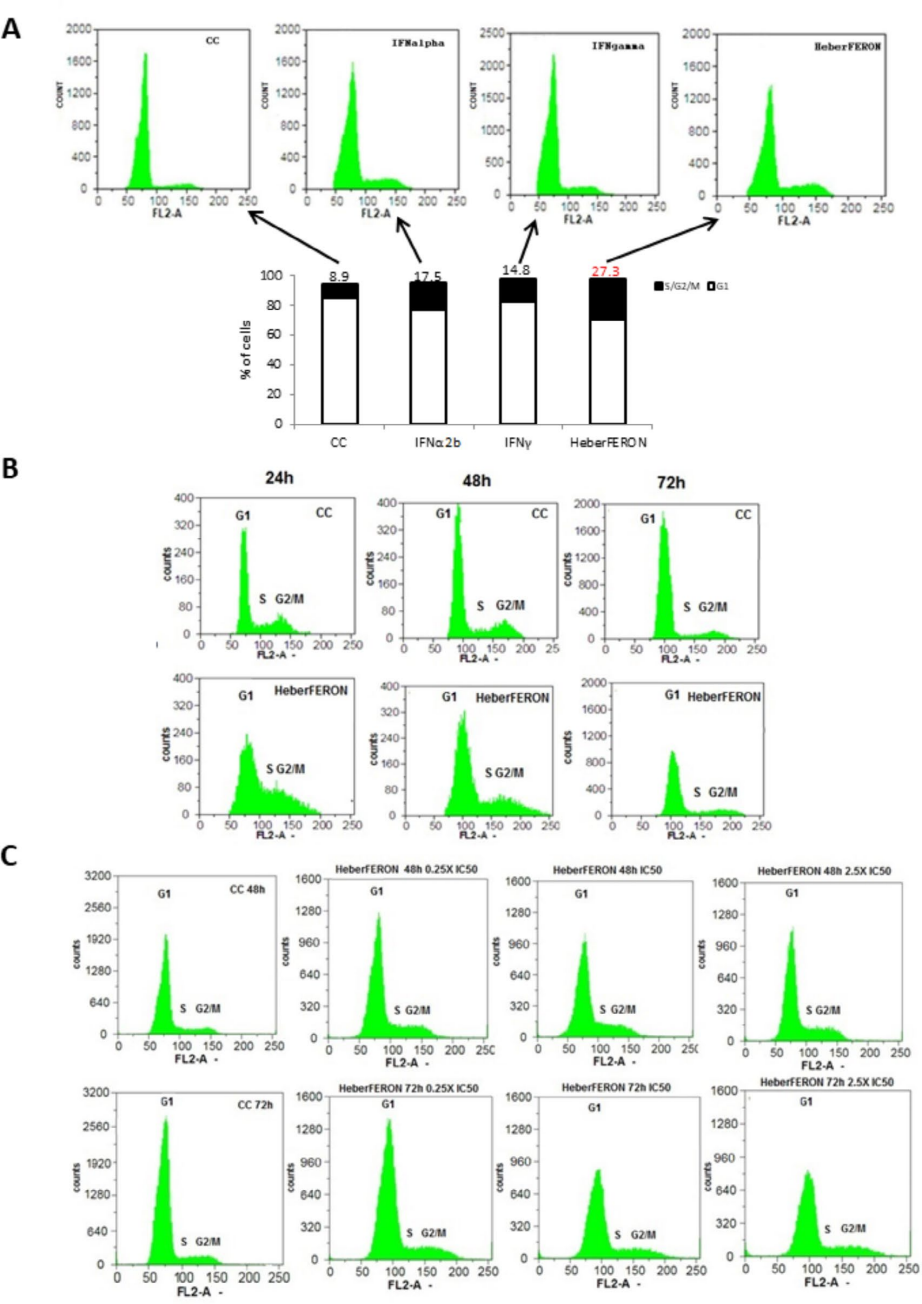
**S-G2/M cell cycle arrest in U-87MG cells by HeberFERON, its dose- and time-dependence**. (**A**) U-87MG cells were pre-incubated with IC50 of HeberFERON and equivalent doses of individual IFNα2b and γ for 72 h and subjected to cell cycle analysis by flow cytometry. The figure shows the counts vs. PI staining in the FL2 channel in the untreated control (CC) compared to the treatment with IFNα2b, IFNγ or HeberFERON in relation to the percentage of cells in phase G1 or S/G2/M. Cell cycle analysis was carried out (**B**) at 24 h, 48 and 72 h of the cell treatment with HeberFERON (IC50 = 4000 IU/mL) and (**C**) after 48 and 72 h of treating the cells with 1000 (0.25XIC50), 4000 (IC50) and 10,000 (2.5XIC50) IU/mL of HeberFERON

We tested HeberFERON to study how it modulates cell cycle dynamics in U-87MG cells, compared to individual IFNα2b and IFNγ (Fig. 9A). HeberFERON induced an S-G2/M cell cycle arrest at 72 h of the treatment using the IC50 dose (27.3% of the cells in these phases compared to 8.9% for the untreated culture), whereas IFNα2b and IFNγ induced a certain arrest but in a smaller percentage of cells (17.5% and 14.8%, respectively). This arrest is observed as early as of 24 h of the treatment (Fig. 9B), at which time the cycle dynamics is highly affected. This effect is dose- and time-dependent as shown in Fig. 9C. The extension of the effect at a dose of 0.25XIC50 (1000 IU/mL) is barely observed but the S-G2/M arrest can be seen at IC50 (4000 IU/mL) and 2.5XIC50 (10,000 IU/mL), with a higher impact at 72 h with the 10,000 IU/mL dose of HeberFERON.

## Discussion

Glioblastoma is one of the most aggressive types of malignant central nervous system tumors, with a rapid infiltrative growth rate and provoking a heterogeneous disease [37]. Although several therapeutic methods have been tested, the progress in the overall survival (OS) rate of GBM is limited [38]. Due to the lack of a truly effective therapy, new approaches for increasing the OS and/or the quality of life of the patients are essential.

The use of IFNs in cancer therapy, including glioma has been reported before with varying results [38, 39], however, HeberFERON has shown promising results in clinical trials [7]. The understanding of its molecular mechanism of action could become a guide for redesigning therapies, suggesting new formulations and combining it with other anti-cancer drugs.

Here, we report the first high throughput transcriptomic analysis of the model human glioblastoma cell line, U-87MG, treated with HeberFERON, IFNα2b and IFNγ for 72 h.

As found previously by Tan et al. [40] and Sanda et al. [4], our experiment showed that the combination of type I and type II IFNs modulates a much larger number of genes than either individual IFN alone.

We showed how multidimensional scaling was able to distinguish samples in each group, but there are common processes and/or genes between groups that are related to the very well established IFN functions in the antiviral, immune and inflammatory responses. Previous studies evidenced that the combination of type I and type II IFNs resulted in the up-regulation of ISGF3 components, enhancing the expression of ISRE and GAS-containing genes associated with a direct antiviral state [40].

Tan *et al* [40] also reported that 26% of the probe sets with differential modulation corresponded to components involved in antigen presentation and processing, immune cell recruitment or complement system function. Several interferon-stimulated genes (ISGs) as those encoding for 2’-5’ OAS, RNaseL, PKR or IRF9 also participate in the antiproliferative effects [41]. These functions have been used in the treatment of viral and neoplastic diseases. In the context of a brain tumor, immune response regulation could also contribute to the overall effect of this product [39].

The enrichment analysis showed similarities and differences between the three treatments with a remarkably distinctive behavior of HeberFERON targeting a range of cell cycle events and more specifically, the mitotic cell cycle. Meta-analysis from several gene expression studies in GBM concluded that mitosis is one of the most relevant biological events in this complex disease [42].

Cell cycle is a highly regulated process at transcription level, by phosphorylation or protein localization changes and degradation. As of G1, cells increase in size, they copy DNA and duplicate centrosomes in S, and get ready (in G2) to divide in mitosis. Surveillance mechanisms through cyclins and cyclin-dependent-kinases (CDK) ensure proper timing of cellular events at G1/S, G2/M and spindle-assembly Checkpoints. Afterwards, the transition toward the prophase, prometaphase, metaphase, anaphase and telophase mitosis stages occurred through an orderly protein degradation by SCF (Skp1/Cullin/F-box) and APC/C protein complexes [43].

Along these stages chromosomes primarily condense, and centrosomes begin to separate in the prophase. The mitotic spindle is formed and the interaction of the microtubules with the spindle and kinetochore protein complexes at the centrosomes, in the prometaphase, will enable the future separation of sister chromatids to the opposite poles of the cells. In the metaphase/anaphase the transition chromosomes are bi-oriented and the spindle checkpoint will ensure the formation of this configuration. Spindle poisons, leading to the mitotic arrest of cancer cells, have encouraged the search for new inhibitors. There are promising ongoing trials for GBM targeting therapy with G2/M inhibitors, including inhibitors of Aurora kinases, PLK1, Survivin, BUB1 and BUBR1 [44].

The events targeted only by HeberFERON include the “Mitotic Prometaphase”, “Metaphase and Anaphases” as well as the “Cell Cycle Checkpoints”. As part of these events, we found genes showing the greatest down-regulation produced by HeberFERON. This is the case of those encoding for prometaphase proteins PLK1, AURKB, BIRC5/Survivin, CCNB1, CENPA, CENPF, ZWINT and proteins participating in the spindle checkpoint as CDC20, BUB1, BUB1R and CENPE. Moreover, the FOXM1 transcription factor network was the most highly enriched pathway by HeberFERON when we applied the CPEA, with a significant down-regulation of the genes involved. This transcription factor plays an essential role in mitotic progression in general [45] and it is critical in GBM development and progression, making it an attractive drug target [46]. The observance of the expression of FOXM1 in multiple samples of four types of brain tumors in the GlioVis data portal clearly shows that the highest expression is found in GBM. The use of HeberFERON may delay the progression of GBMs and it may help reduce the resistance to the temozolomide (TMZ) treatment. TMZ is the standard chemotherapy for GBM since 2005 [47] and when combined with radiotherapy, it offers a two-month increase in the OS as average [48]. GBM therapy failure can be due to TMZ resistance, enhanced by the CXCL12/CXCR4 promotion of migration of GBM cells by up-regulating FOXM1. Thus, FOXM1 silencing can partially reverse this resistance [49]. The regulation of mRNAs expression by FOXM1 enables the accumulation of cyclin B1 during G2 and a decrease after mitosis. FOXM1 also controls AURKB, CENPA, CENPF, NEK2, PLK1 and Survivin. Most of these genes are commonly overexpressed in different types of human cancer, including GBM [50–53], which was also shown in the GlioVis portal analysis of brain tumor samples.

AURKB and Survivin, together with Borealin and INCENP, form the chromosomal passenger complex that localizes at the kinetochores and chromosomes during early mitosis and operates in the microtubule–kinetochore interactions, sister chromatid cohesion, and the spindle-assembly checkpoint [54].

Aurora kinases regulate different aspects of cell division. Aurora kinase B (AURKB) is present in the centromeres in prophase and metaphase and it is located in the central mitotic spindle, and is crucial for the segregation of chromosomes and cytokinesis [55]. The expressions of AURK A and B and kinase activities are high in a variety of human cancers and they are associated with high levels of proliferation and poor prognosis [56]. AURKB was proposed as a prognosis marker for GBM from a study that showed an overexpression in 25 GBM samples and a correlation of its expression levels with survival [53]. Small molecules inhibitors of Aurora kinases A and B interfere with the centrosome function during mitosis and they disrupt the assembly point of the mitotic spindle resulting in the polyploidization and apoptosis of proliferating cells [57, 58]. The strong down-regulation of AURKB by HeberFERON may contribute to the increase in the OS of GBM patients.

Survivin (BIRC5) acts as an apoptosis suppressor, and it plays a central role in cell division. It is expressed in the G2/M phase, and is located in the mitotic spindle interacting with tubulin, while also playing a role in the regulation of mitosis. It was also reported to be located in the centromeres, influencing the stability of the kinetochore-microtubule junction and in the control signal of the mitotic spindle, physically interacting with AURKB [59]. It is also highly expressed in most cancers. Hence, in some subtypes, it has a prognostic value related to antineoplastic resistance and radiotherapy as occurring with cisplatin [60]. Several studies have shown that the inhibition of Survivin reduces tumor growth, it increases apoptosis and sensitizes the tumor to different chemotherapeutic agents [61, 62]. Hence, the inhibition of BIRC5 by HeberFERON suggests its possible combination with several chemotherapeutic agents (vincristine, cisplatin, bortezomib, tamoxifen) to avoid resistance. This protein was found to be essential for cell proliferation but it is not required for the survival of normal cells [63]. According to Beardmore et al. [59] BIRC5 regulation appeared to be linked to the p53 protein.

The PLKs belong to a family of Serine-Threonine kinases that play key roles in the control of the cell cycle and the response to DNA damage. In the G2/M checkpoint, cells with damaged DNA are prevented from starting mitosis. The activity of the cyclin B-CDK1 complex is essential at this point, and a feedback amplification loop is established among Aurora kinase A, PLK1 and CDK1 by phosphorylation. Many studies have shown PLK1 inhibition leading to the death of cancer cells by interfering with multiple stages of mitosis [64]. PLK1 mRNA expression strongly correlated with WHO grades, KPS and the recurrence of tumors of patients with gliomas. The down-regulation of PLK1 at both mRNA and protein levels was able to inhibit growth, induce the arrest of the cell cycle in G2/M and increase glioma cell apoptosis [65]. PLK1 promotes the translocation of cyclin B into the nucleus during the prophase and initiates cycling by activating CDC25 phosphatase and inactivating WEE1/MYT1 kinases. Activated cyclin B–CDK1 stimulates the activity of APC/C CDC20 through the phosphorylation of several subunits of APC/C and CDC20 but PLK1 also phosphorylates and activates APC/C. Besides the role of the cyclin B-CDK1 complex at the G2/M checkpoint, its activity also contributes to the inactivation of the mitotic spindle checkpoint. The loss or low expression of cyclin B1 (CCNB1) causes a deficient binding between the kinetochores and the microtubules, defects in the alignment of the chromosomes and it delays the start of the anaphase [66]. HeberFERON causes a significant decrease in CCNB1, which distinguishes it from the effects of the individual IFNs. This could contribute to the impairment of the start of the anaphase and cell cycle arrest.

Moreover, results of GSEA show that the function of CENPA at the centromere and kinesins could also be involved in the molecular mechanism of HeberFERON. CENPA is required for the kinetochore recruitment of all other kinetochore components. The phosphorylation of CENPA by AURKB plays an important role in cytokinesis [55], while Ser7 phosphorylation by AURKA is required for the concentration of AURKB at the centromeres and for the functioning of the kinetochore [67]. HJRUP interacts with CENPA and it is required for its centromeric assembly and deposition [68, 69]. Here, CENPA, HJURP and the inner kinetochore components, CENPM and CENPP, showed decreased expressions due to HeberFERON. Kinesins are crucial at different stages of cell division. Particularly, KIF23 and KIF20A play a major role in cytokinesis [70]. KIF23 is responsible for bundling and stabilizing the microtubules, it requires INCENP for its recruitment [71] and it is regulated by CDK1, Aurora B and PLK1 [72, 73]. The down-regulation of KIF23 was shown to suppress glioma proliferation, and it was therefore proposed as a potential therapeutic target for GBM [74]. PLK1 directly phosphorylates KIF20A and regulates its motor properties, and at the same time, KIF20A seems to be essential for the normal localization of PLK1 at the central spindle [75]. HeberFERON down-regulates a set of kinesins including KIF23, KIF20A, KIF11, KIF22 and KIFC1 that could affect their motor functions.

The spindle checkpoint is activated by either the presence of unattached kinetochores or the absence of tension between the paired kinetochores. Kinetochores couple sister chromatids to dynamic microtubules during congression and anaphase; this allows their separation and partitioning to the daughter cells [76]. Unattached kinetochores attract several components of the checkpoint sensors (including BUB1, BUBR1, CENPE and MAD2), catalyzing the formation of mitotic checkpoint complexes (in the outer kinetochore), resulting in the inhibition APC/C-CDC20.

It is also evident that HeberFERON induced a marked decrease of genes encoding checkpoint mitotic complex components, such as BUB1, BUB1R, CENPE, CENPF and CDC20. Morrow et al. [77] suggested that checkpoint is composed of one arm dependent on BUB1 and the other on AURKB, both converging at the mitotic checkpoint complex [77]. Furthermore, BUB1R kinase is regulated by CENPE and, at the same time, it regulates the APC/C-CDC20 proteolytic machinery. The knockdown of BUB1B/BUBR1 inhibited the expansion of brain tumor–initiating cell isolates, both in vitro and in vivo, without affecting the proliferation of the human neural stem cells or astrocytes [78]. These results distinguish this protein as the top-scoring lethal glioblastoma kinase. HeberFERON then target multiple transcripts for proteins that are important in passing this control point in the cell cycle.

Zhou et al. [79] found CDC20, TOP2A and PBK to be highly up-regulated in glioblastoma samples compared with healthy tissue. These genes were all identified as hub genes in DEGs network and inhibited by HeberFERON. A Bisogenet network was built with the immediate neighbors of CDC20, TOP2A and PBK containing 275 highly connected nodes, where CDC20, P53 and TOP2A showed the highest degree, in that order.

TTK was identified as the most up-regulated gene encoding protein kinase in glioma stem-like cells. It was essential for clonogenicity and tumor propagation correlating with poor prognosis in GBM patients [80]. HeberFERON also down-regulates TTK. These genes together with ZWINT, HJURP, CENPA and several kinesins are connected in a network from the cascade initiators, STAT1&2, passing through the PKR and ATF3 nodes; all these transcription factors highly increased with HeberFERON.

Consistent with the above interpretation, cell cycle FACS analysis showed the dose- and time-dependent effect of HeberFERON on the process, with a clear effect of an arrest as of 24 h of the treatment. At this time point, cells accumulate at the S/G2/M stages. At 72 h of the treatment, the time point selected for the transcriptomic experiment, the percentage of cells at the S/G2/M stages in the HeberFERON group exceeded in 10% and 13% the groups treated with IFNγ and α2b, respectively. Altogether, this ensures an improbable cycling beyond the anaphase or ultimately, the impairment of cycle completion and cytokinesis.

**Fig. 10:**
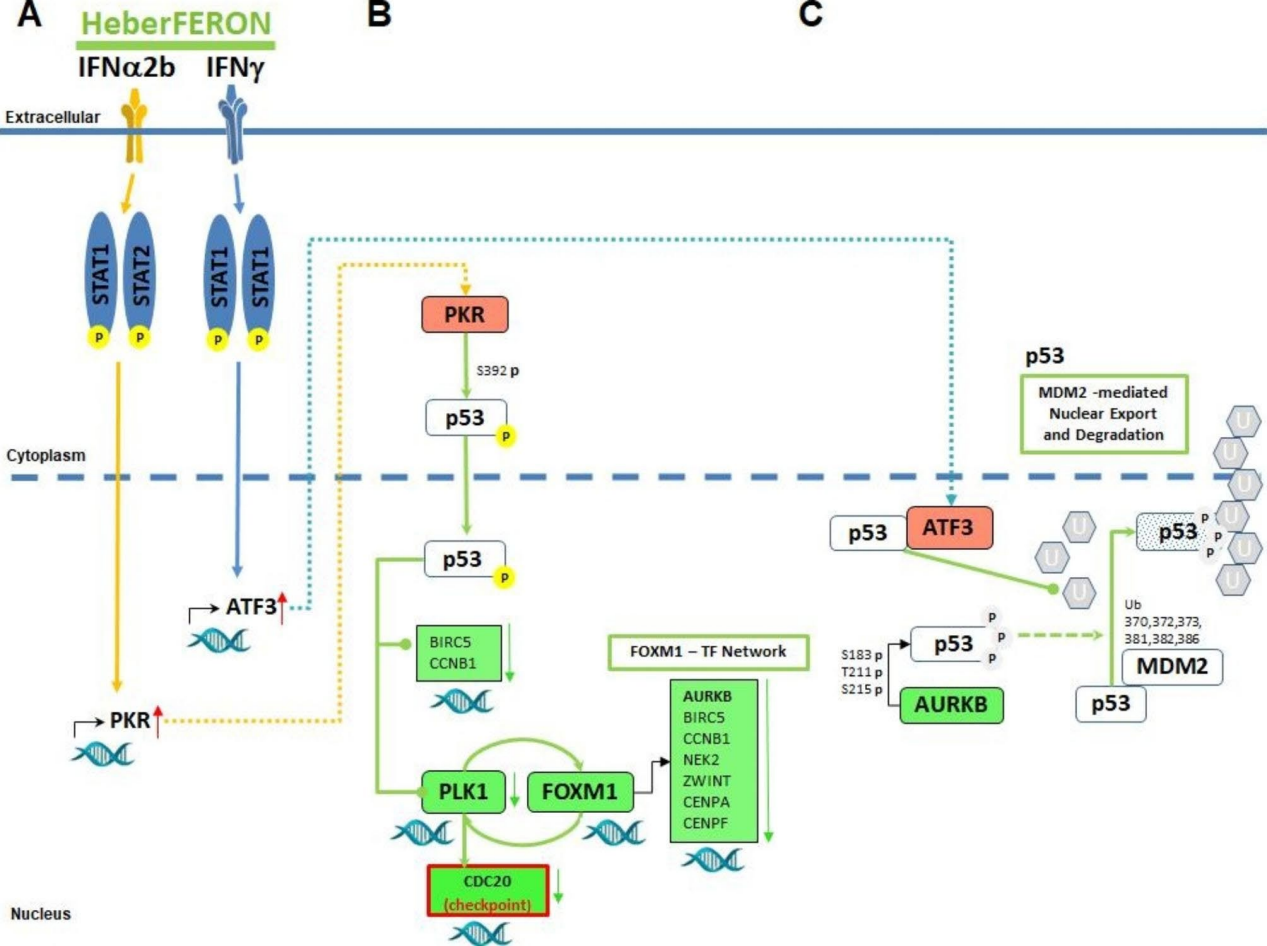
**Model proposal of molecular mechanisms involved in the distinctive action of HeberFERON**. In this schematic representation, HeberFERON activates PKR and ATF3 simultaneously through the STAT complex. (**A**) IFNα2b and IFNγ induce the up-regulation of PKR and ATF3, respectively. (**B**) PKR phosphorylates p53 at Serine 392; p53 is translocated to the nucleus where it represses the expression of PLK1; consequently, FOXM1 phosphorylation is reduced and the FOXM1 transcription network is down-regulated. CDC20, also activated by PLK1, is down-regulated, delaying the exit from mitosis. (**C**) AURKB, as part of the FOXM1 transcription network, is down-regulated and its phosphorylation over p53 at residues Ser183, Thr211, and Ser215 is reduced, slowing down p53 degradation through MDM2-mediated ubiquitination. In parallel ATF3 up-regulation increases its association with p53 while also reducing MDM2 ubiquitination of p53 and its export from the nucleus to the cytoplasm. Rounded rectangles filled in red and green represent up-regulated and down-regulated gene/proteins, respectively. The letter “p” in a yellow circle represents phosphorylated residues; the letter “p” in a gray circle represents the residue with diminished phosphorylation. The letter “U” in a gray hexagon represents the ubiquitin protein slowly added into the target protein, p53

Taking into account these elements we propose a general model explaining the distinctive effect of HeberFERON in cell cycle in U-87MG (Fig. 10). It is based on the simultaneous activation of the transcriptional factor ATF3 and PKR/EIF2AK2. PKR is significantly up-regulated in IFNα2b and HeberFERON samples, while ATF3 is up-regulated in IFNγ and HeberFERON. That is, the activation of both proteins only occurs in samples treated with HeberFERON. Moreover, both proteins are functionally related to p53 as it is shown in the networks. In this context the wildtype status of p53 in the U-87MG cell line gains relevance for its known tumor suppression function, in which PKR plays an important role [81]. It phosphorylates p53 at Ser392 [82] and this phosphorylation is important for p53 activation and localization [83]. The second element of the model relies on the role of ATF3 activation on blocking the MDM2 degradation of p53 [84] and preventing the translocation of p53 to the cytoplasm, thus contributing to its tumor-suppressor activity [85]. In glioblastomas, p53 is known to be located mostly in the cytoplasm [86]. Its localization in the nucleus is associated to a longer survival of GBM patients [87]. Sequestration of p53 in the cytoplasm prevents its translocation to the nucleus and presumably avoids its suppressive function. This was also found in poorly differentiated pediatric neuroblastomas [88]. Furthermore, in primary GBM the location of the p53 wild type in the cytoplasm was correlated to the expression of vimentin [89]. These elements, together with the known functions of ATF3 and PKR, suggest further studies on the location of p53 in cells treated with HeberFERON. The genes repressed by p53 include CCNB1 and BIRC5, both genes are strongly down-regulated by HeberFERON. An additional mechanism includes the p53-mediated increased expression of GADD45 that binds to CDK1 and prevents the formation of the cyclinB-CDK1 complex and G2 arrest [90]. The increase of GADD45 and decrease of CCNB1 expressions, mediated by p53, can be contributing to cycle arrest.

In the model of Fig. 10 we also show the FOXM1 network, which is down-regulated by HeberFERON. Phosphorylation of FOXM1 by PLK1 provides a positive feedback loop that is essential for mitotic progression [91]. APC/C-CDC20 complex components must also be phosphorylated by PLK1 to contribute to the progression of mitosis stages by sequential protein degradation [92]. The APC/C-CDC20 function will also depend on the mitotic spindle checkpoint complex. Additionally, Aurora kinase B is a negative regulator of p53 by the phosphorylation on Ser183, Thr211, and Ser215, which contributes to p53 degradation through MDM2-mediated ubiquitination [93]. All these facts are integrated into the proposed model.

Diagnostic, prognostic or predictive molecular biomarkers are limited in GBM although they could have an impact in the OS and in the personalization of treatments [94]. The best known molecular biomarker is the methylation status of O(6)-methylguanine-DNA-methyltransferase (MGMT) as a predictor of TMZ [95] and radiation resistance [96]. Other authors have described IFNβ or IFNγ associated gene signatures to predict OS, efficacy of immunotherapy and radiotherapy among glioblastoma patients [97, 98]. Eventually, these genes signatures should be validated in clinical practice.

Here, we identified a signature of poor prognosis in the Proneural subtype of GBM [99]. The expression levels of genes belonging to this poor prognosis signature increased with the HeberFERON treatment as well as with the IFNα2b treatment. The reformulation of HeberFERON with different IFNα2b and γ proportions could become an alternative to treat this subtype of GBM tumors. This proposal must also be validated.

## Conclusions

As part of this investigation we described the transcriptomic profile of HeberFERON compared to individual IFN treatments. It is highlighted that, as in other IFNs combinations, HeberFERON highly stimulates the transcription of genes involved in antiviral and immune responses. Of greater interest was to find that HeberFERON distinctively targets transcripts encoding proteins that participate in cell cycle events. As part of these events, we found key players in the mitotic prometaphase to anaphase stages, the spindle checkpoint and proteolytic degradation in mitosis by APC/C-CDC20, which could explain the G2/M arrest and anti-proliferation effect on U-87MG. Signaling from the STATs through PKR and ATF3 factors converge at p53, and the signal propagates from this cascade hub to the cell cycle and proliferation players, particularly in GBM, in a multi-targeted way. These findings support our proposed general mechanistic model and also underscore the use of HeberFERON alone or combined with chemotherapeutics in the treatment of GBM.

### Electronic supplementary material

Below is the link to the electronic supplementary material.

Supplementary Material 1

Supplementary Material 2

Supplementary Material 3

## Data Availability

The data discussed in this publication have been deposited in NCBI’s Gene Expression Omnibus Edgar et al. [100] and are accessible through GEO Series accession number GSE214832 (https://www.ncbi.nlm.nih.gov/geo/query/acc.cgi?acc=GSExxx).

## Abbreviations

CDK: Cyclin Dependent Kinase
CPEA: Comparative Pathway Enrichment Analysis
FDR: False Discovery Rate
GBM: Glioblastoma
GO: Gene Ontology
GSEA: Gene Set Enrichment Analysis
NES: Normalized Enrichment Score
OS: Overall Survival
qRT-PCR: Quantitative reverse transcriptase PCR

## References

[1] Zsakai L, Sipos A, Dobos J, Eros D, Szantai-Kis C, Banhegyi P, Pato J, Orfi L, Matula Z, Mikala G (2019). Targeted drug combination therapy design based on driver genes. Oncotarget.

[2] Morikawa K, Kubagawa H, Suzuki T, Cooper MD (1987). Recombinant interferon-alpha, -beta, and -gamma enhance the proliferative response of human B cells. J Immunol.

[3] Sainz B, Mossel EC, Peters CJ, Garry RF (2004). Interferon-beta and interferon-gamma synergistically inhibit the replication of severe acute respiratory syndrome-associated coronavirus (SARS-CoV). Virology.

[4] Sanda C, Weitzel P, Tsukahara T, Schaley J, Edenberg HJ, Stephens MA, McClintick JN, Blatt LM, Li L, Brodsky L (2006). Differential gene induction by type I and type II interferons and their combination. J Interferon Cytokine Research: Official J Int Soc Interferon Cytokine Res.

[5] Thomas DC, Samuel CE (1992). Mechanism of interferon action: alpha and gamma interferons differentially affect mRNA levels of the catalytic subunit of protein kinase A and protein mx in human cells. J Virol.

[6] Bello-Rivero I, Garcia-Vega Y, Valenzuela-Silva C, Bello-Alvarez C, Vázquez-Blomquist D, Lopez-Saura P (2013). Development of a new formulation of interferons (HEBERPAG) for BCC treatment. J Cancer Res Therapy.

[7] Bello-Rivero I, Garcia-Vega Y, Duncan-Roberts Y, Vazquez-Blomquistc D, Santana-Milian H, Besada-Perez V, Rios-Cabrera M (2018). HeberFERON, a new formulation of IFNs with improved pharmacodynamics: perspective for cancer treatment. Semin Oncol.

[8] Garcia-Vega Y, Salva-Camaño S, García-Iglesias E, Cubero-Rego D, González-Gonzalez J, I B-R (2015). CIGB-128, as compassionate intracranial treatment in patients with non-operable or progressive high grade gliomas. J Cancer Res Therapy.

[9] Platanias LC (2005). Mechanisms of type-I- and type-II-interferon-mediated signalling. Nat Rev Immunol.

[10] Taniguchi T, Takaoka A (2001). A weak signal for strong responses: interferon-alpha/beta revisited. Nat Rev Mol Cell Biol.

[11] Shi W, Oshlack A, Smyth GK (2010). Optimizing the noise versus bias trade-off for Illumina whole genome expression BeadChips. Nucleic Acids Res.

[12] Ritchie ME, Phipson B, Wu D, Hu Y, Law CW, Shi W, Smyth GK (2015). Limma powers differential expression analyses for RNA-sequencing and microarray studies. Nucleic Acids Res.

[13] Phipson B, Lee S, Majewski IJ, Alexander WS, Smyth GK (2016). Robust hyperparameter estimation protects against Hypervariable genes and improves power to detect Differential expression. The Annals of Applied Statistics.

[14] Zar JH. Biostatistical Analysis. 4th ed. Prentice Hall, NJ. edn; 1999.

[15] Benjamini Y, Drai D, Elmer G, Kafkafi N, Golani I (2001). Controlling the false discovery rate in behavior genetics research. Behav Brain Res.

[16] Saeed AI, Bhagabati NK, Braisted JC, Liang W, Sharov V, Howe EA, Li J, Thiagarajan M, White JA, Quackenbush J (2006). TM4 microarray software suite. Methods Enzymol.

[17] Saeed AI, Sharov V, White J, Li J, Liang W, Bhagabati N, Braisted J, Klapa M, Currier T, Thiagarajan M (2003). TM4: a free, open-source system for microarray data management and analysis. Biotechniques.

[18] Chen J, Bardes EE, Aronow BJ, Jegga AG. ToppGene suite for gene list enrichment analysis and candidate gene prioritization. Nucleic Acids Res 2009, 37(Web Server issue):W305–311. 10.1093/nar/gkp427 PMC2703978 19465376

[19] Carmona-Saez P, Chagoyen M, Tirado F, Carazo JM, Pascual-Montano A (2007). GENECODIS: a web-based tool for finding significant concurrent annotations in gene lists. Genome Biol.

[20] Nogales-Cadenas R, Carmona-Saez P, Vazquez M, Vicente C, Yang X, Tirado F, Carazo JM, Pascual-Montano A. GeneCodis: interpreting gene lists through enrichment analysis and integration of diverse biological information. Nucleic Acids Res 2009, 37(Web Server issue):W317–322. 10.1093/nar/gkp416 PMC2703901 19465387

[21] Dennis G, Sherman BT, Hosack DA, Yang J, Gao W, Lane HC, Lempicki RA (2003). DAVID: database for annotation, visualization, and Integrated Discovery. Genome Biol.

[22] Huang da W, Sherman BT, Lempicki RA (2009). Bioinformatics enrichment tools: paths toward the comprehensive functional analysis of large gene lists. Nucleic Acids Res.

[23] Huang R, Grishagin I, Wang Y, Zhao T, Greene J, Obenauer JC, Ngan D, Nguyen DT, Guha R, Jadhav A (2019). The NCATS BioPlanet - An Integrated platform for exploring the Universe of Cellular Signaling Pathways for Toxicology, Systems Biology, and Chemical Genomics. Front Pharmacol.

[24] Ashburner M, Ball CA, Blake JA, Botstein D, Butler H, Cherry JM, Davis AP, Dolinski K, Dwight SS, Eppig JT (2000). Gene ontology: tool for the unification of biology. The Gene Ontology Consortium. Nat Genet.

[25] Kanehisa M, Araki M, Goto S, Hattori M, Hirakawa M, Itoh M, Katayama T, Kawashima S, Okuda S, Tokimatsu T (2008). KEGG for linking genomes to life and the environment. Nucleic Acids Res.

[26] Ogata H, Goto S, Sato K, Fujibuchi W, Bono H, Kanehisa M (1999). KEGG: Kyoto Encyclopedia of genes and genomes. Nucleic Acids Res.

[27] Croft D, Mundo AF, Haw R, Milacic M, Weiser J, Wu G, Caudy M, Garapati P, Gillespie M, Kamdar MR (2014). The Reactome pathway knowledgebase. Nucleic Acids Res.

[28] Fabregat A, Sidiropoulos K, Garapati P, Gillespie M, Hausmann K, Haw R, Jassal B, Jupe S, Korninger F, McKay S (2016). The Reactome pathway knowledgebase. Nucleic Acids Res.

[29] Schaefer CF, Anthony K, Krupa S, Buchoff J, Day M, Hannay T, Buetow KH (2009). PID: the Pathway Interaction Database. Nucleic Acids Res.

[30] Subramanian A, Tamayo P, Mootha VK, Mukherjee S, Ebert BL, Gillette MA, Paulovich A, Pomeroy SL, Golub TR, Lander ES (2005). Gene set enrichment analysis: a knowledge-based approach for interpreting genome-wide expression profiles. Proc Natl Acad Sci USA.

[31] Martin A, Ochagavia ME, Rabasa LC, Miranda J, Fernandez-de-Cossio J, Bringas R (2010). BisoGenet: a new tool for gene network building, visualization and analysis. BMC Bioinformatics.

[32] Bowman RL, Wang Q, Carro A, Verhaak RG, Squatrito M (2017). GlioVis data portal for visualization and analysis of brain tumor expression datasets. Neurooncology.

[33] Vazquez-Blomquist D, Fernandez JR, Miranda J, Bello C, Silva JA, Estrada RC, Novoa LI, Palenzuela D, Bello I (2012). Selection of reference genes for use in quantitative reverse transcription PCR assays when using interferons in U87MG. Mol Biol Rep.

[34] Pfaffl MW (2001). A new mathematical model for relative quantification in real-time RT-PCR. Nucleic Acids Res.

[35] Pfaffl MW, Horgan GW, Dempfle L (2002). Relative expression software tool (REST) for group-wise comparison and statistical analysis of relative expression results in real-time PCR. Nucleic Acids Res.

[36] Clark MJ, Homer N, O’Connor BD, Chen Z, Eskin A, Lee H, Merriman B, Nelson SF (2010). U87MG decoded: the genomic sequence of a cytogenetically aberrant human cancer cell line. PLoS Genet.

[37] Ostrom QT, Cioffi G, Waite K, Kruchko C, Barnholtz-Sloan JS (2021). CBTRUS Statistical Report: primary brain and other Central Nervous System Tumors diagnosed in the United States in 2014–2018. Neurooncology.

[38] Janjua TI, Rewatkar P, Ahmed-Cox A, Saeed I, Mansfeld FM, Kulshreshtha R, Kumeria T, Ziegler DS, Kavallaris M, Mazzieri R (2021). Frontiers in the treatment of glioblastoma: past, present and emerging. Adv Drug Deliv Rev.

[39] Dapash M, Castro B, Hou D, Lee-Chang C. Current Immunotherapeutic Strategies for the Treatment of Glioblastoma. *Cancers* 2021, 13(18). 10.3390/cancers13184548 PMC8467991 34572775

[40] Tan H, Derrick J, Hong J, Sanda C, Grosse WM, Edenberg HJ, Taylor M, Seiwert S, Blatt LM (2005). Global transcriptional profiling demonstrates the combination of type I and type II interferon enhances antiviral and immune responses at clinically relevant doses. J Interferon Cytokine Research: Official J Int Soc Interferon Cytokine Res.

[41] Bekisz J, Baron S, Balinsky C, Morrow A, Zoon KC (2010). Antiproliferative Properties of type I and type II Interferon. Pharmaceuticals.

[42] Horvath S, Zhang B, Carlson M, Lu KV, Zhu S, Felciano RM, Laurance MF, Zhao W, Qi S, Chen Z (2006). Analysis of oncogenic signaling networks in glioblastoma identifies ASPM as a molecular target. Proc Natl Acad Sci USA.

[43] Poon RY (2016). Cell cycle control: a system of interlinking oscillators. Methods Mol Biol.

[44] Castro-Gamero AM, Pezuk JA, Brassesco MS, Tone LG (2018). G2/M inhibitors as pharmacotherapeutic opportunities for glioblastoma: the old, the new, and the future. Cancer Biology & Medicine.

[45] Wang IC, Chen YJ, Hughes D, Petrovic V, Major ML, Park HJ, Tan Y, Ackerson T, Costa RH (2005). Forkhead box M1 regulates the transcriptional network of genes essential for mitotic progression and genes encoding the SCF (Skp2-Cks1) ubiquitin ligase. Mol Cell Biol.

[46] Wang Z, Zhang S, Siu TL, Huang S (2015). Glioblastoma multiforme formation and EMT: role of FoxM1 transcription factor. Curr Pharm Design.

[47] Singh N, Miner A, Hennis L, Mittal S (2021). Mechanisms of temozolomide resistance in glioblastoma - a comprehensive review. Cancer drug Resistance.

[48] Stupp R, Mason WP, van den Bent MJ, Weller M, Fisher B, Taphoorn MJ, Belanger K, Brandes AA, Marosi C, Bogdahn U (2005). Radiotherapy plus concomitant and adjuvant temozolomide for glioblastoma. N Engl J Med.

[49] Wang S, Chen C, Li J, Xu X, Chen W, Li F (2020). The CXCL12/CXCR4 axis confers temozolomide resistance to human glioblastoma cells via up-regulation of FOXM1. J Neurol Sci.

[50] Alafate W, Wang M, Zuo J, Wu W, Sun L, Liu C, Xie W, Wang J (2019). Targeting Aurora kinase B attenuates chemoresistance in glioblastoma via a synergistic manner with temozolomide. Pathol Res Pract.

[51] Cheng MW, Wang BC, Weng ZQ, Zhu XW (2012). Clinicopathological significance of Polo-like kinase 1 (PLK1) expression in human malignant glioma. Acta Histochem.

[52] Tong X, Yang P, Wang K, Liu Y, Liu X, Shan X, Huang R, Zhang K, Wang J (2019). Survivin is a prognostic indicator in glioblastoma and may be a target of microRNA-218. Oncol Lett.

[53] Zeng WF, Navaratne K, Prayson RA, Weil RJ (2007). Aurora B expression correlates with aggressive behaviour in glioblastoma multiforme. J Clin Pathol.

[54] Honda R, Korner R, Nigg EA (2003). Exploring the functional interactions between Aurora B, INCENP, and survivin in mitosis. Mol Biol Cell.

[55] Zeitlin SG, Shelby RD, Sullivan KF (2001). CENP-A is phosphorylated by Aurora B kinase and plays an unexpected role in completion of cytokinesis. J Cell Biol.

[56] Sankhe K, Prabhu A, Khan T (2021). Design strategies, SAR, and mechanistic insight of Aurora kinase inhibitors in cancer. Chem Biol Drug Des.

[57] Li J, Anderson MG, Tucker LA, Shen Y, Glaser KB, Shah OJ (2009). Inhibition of Aurora B kinase sensitizes a subset of human glioma cells to TRAIL concomitant with induction of TRAIL-R2. Cell Death Differ.

[58] Tang A, Gao K, Chu L, Zhang R, Yang J, Zheng J (2017). Aurora kinases: novel therapy targets in cancers. Oncotarget.

[59] Beardmore VA, Ahonen LJ, Gorbsky GJ, Kallio MJ (2004). Survivin dynamics increases at centromeres during G2/M phase transition and is regulated by microtubule-attachment and Aurora B kinase activity. J Cell Sci.

[60] Zaffaroni N, Daidone MG (2002). Survivin expression and resistance to anticancer treatments: perspectives for new therapeutic interventions. Drug Resist Updates: Reviews Commentaries Antimicrob Anticancer Chemother.

[61] Cheung CH, Huang CC, Tsai FY, Lee JY, Cheng SM, Chang YC, Huang YC, Chen SH, Chang JY (2013). Survivin - biology and potential as a therapeutic target in oncology. OncoTargets and Therapy.

[62] Mita AC, Mita MM, Nawrocki ST, Giles FJ (2008). Survivin: key regulator of mitosis and apoptosis and novel target for cancer therapeutics. Clin cancer Research: Official J Am Association Cancer Res.

[63] Li D, Hu C, Li H (2018). Survivin as a novel target protein for reducing the proliferation of cancer cells. Biomedical Rep.

[64] Danovi D, Folarin A, Gogolok S, Ender C, Elbatsh AM, Engstrom PG, Stricker SH, Gagrica S, Georgian A, Yu D (2013). A high-content small molecule screen identifies sensitivity of glioblastoma stem cells to inhibition of polo-like kinase 1. PLoS ONE.

[65] Pezuk JA, Brassesco MS, Morales AG, de Oliveira JC, de Paula Queiroz RG, Machado HR, Carlotti CG, Neder L, Scrideli CA, Tone LG (2013). Polo-like kinase 1 inhibition causes decreased proliferation by cell cycle arrest, leading to cell death in glioblastoma. Cancer Gene Ther.

[66] Chen Q, Zhang X, Jiang Q, Clarke PR, Zhang C (2008). Cyclin B1 is localized to unattached kinetochores and contributes to efficient microtubule attachment and proper chromosome alignment during mitosis. Cell Res.

[67] Kunitoku N, Sasayama T, Marumoto T, Zhang D, Honda S, Kobayashi O, Hatakeyama K, Ushio Y, Saya H, Hirota T (2003). CENP-A phosphorylation by Aurora-A in prophase is required for enrichment of Aurora-B at inner centromeres and for kinetochore function. Dev Cell.

[68] Foltz DR, Jansen LE, Bailey AO, Yates JR, Bassett EA, Wood S, Black BE, Cleveland DW (2009). Centromere-specific assembly of CENP-a nucleosomes is mediated by HJURP. Cell.

[69] Zasadzinska E, Barnhart-Dailey MC, Kuich PH, Foltz DR (2013). Dimerization of the CENP-A assembly factor HJURP is required for centromeric nucleosome deposition. EMBO J.

[70] Rath O, Kozielski F (2012). Kinesins and cancer. Nat Rev Cancer.

[71] Zhu C, Bossy-Wetzel E, Jiang W (2005). Recruitment of MKLP1 to the spindle midzone/midbody by INCENP is essential for midbody formation and completion of cytokinesis in human cells. Biochem J.

[72] Lee KS, Yuan YL, Kuriyama R, Erikson RL (1995). Plk is an M-phase-specific protein kinase and interacts with a kinesin-like protein, CHO1/MKLP-1. Mol Cell Biol.

[73] Liu X, Zhou T, Kuriyama R, Erikson RL (2004). Molecular interactions of Polo-like-kinase 1 with the mitotic kinesin-like protein CHO1/MKLP-1. J Cell Sci.

[74] Takahashi S, Fusaki N, Ohta S, Iwahori Y, Iizuka Y, Inagawa K, Kawakami Y, Yoshida K, Toda M (2012). Downregulation of KIF23 suppresses glioma proliferation. J Neurooncol.

[75] Neef R, Preisinger C, Sutcliffe J, Kopajtich R, Nigg EA, Mayer TU, Barr FA (2003). Phosphorylation of mitotic kinesin-like protein 2 by polo-like kinase 1 is required for cytokinesis. J Cell Biol.

[76] Musacchio A, Desai A. A Molecular View of Kinetochore Assembly and function. Biology 2017, 6(1). 10.3390/biology6010005 PMC5371998 28125021

[77] Morrow CJ, Tighe A, Johnson VL, Scott MI, Ditchfield C, Taylor SS (2005). Bub1 and aurora B cooperate to maintain BubR1-mediated inhibition of APC/CCdc20. J Cell Sci.

[78] Ding Y, Hubert CG, Herman J, Corrin P, Toledo CM, Skutt-Kakaria K, Vazquez J, Basom R, Zhang B, Risler JK (2013). Cancer-Specific requirement for BUB1B/BUBR1 in human brain tumor isolates and genetically transformed cells. Cancer Discov.

[79] Zhou Y, Yang L, Zhang X, Chen R, Chen X, Tang W, Zhang M. Identification of Potential Biomarkers in Glioblastoma through Bioinformatic Analysis and Evaluating Their Prognostic Value. *BioMed research international* 2019, 2019:6581576. 10.1155/2019/6581576 PMC6500689 31119182

[80] Wang J, Xie Y, Bai X, Wang N, Yu H, Deng Z, Lian M, Yu S, Liu H, Xie W (2018). Targeting dual specificity protein kinase TTK attenuates tumorigenesis of glioblastoma. Oncotarget.

[81] Yoon CH, Lee ES, Lim DS, Bae YS (2009). PKR, a p53 target gene, plays a crucial role in the tumor-suppressor function of p53. Proc Natl Acad Sci USA.

[82] Cuddihy AR, Wong AH, Tam NW, Li S, Koromilas AE (1999). The double-stranded RNA activated protein kinase PKR physically associates with the tumor suppressor p53 protein and phosphorylates human p53 on serine 392 in vitro. Oncogene.

[83] Castrogiovanni C, Waterschoot B, De Backer O, Dumont P (2018). Serine 392 phosphorylation modulates p53 mitochondrial translocation and transcription-independent apoptosis. Cell Death Differ.

[84] Yan C, Lu D, Hai T, Boyd DD (2005). Activating transcription factor 3, a stress sensor, activates p53 by blocking its ubiquitination. EMBO J.

[85] Lohrum MA, Woods DB, Ludwig RL, Balint E, Vousden KH (2001). C-terminal ubiquitination of p53 contributes to nuclear export. Mol Cell Biol.

[86] Nagpal J, Jamoona A, Gulati ND, Mohan A, Braun A, Murali R, Jhanwar-Uniyal M (2006). Revisiting the role of p53 in primary and secondary glioblastomas. Anticancer Res.

[87] Burton EC, Lamborn KR, Forsyth P, Scott J, O’Campo J, Uyehara-Lock J, Prados M, Berger M, Passe S, Uhm J (2002). Aberrant p53, mdm2, and proliferation differ in glioblastomas from long-term compared with typical survivors. Clin Cancer Res.

[88] Moll UM, LaQuaglia M, Benard J, Riou G (1995). Wild-type p53 protein undergoes cytoplasmic sequestration in undifferentiated neuroblastomas but not in differentiated tumors. Proc Natl Acad Sci USA.

[89] Sembritzki O, Hagel C, Lamszus K, Deppert W, Bohn W (2002). Cytoplasmic localization of wild-type p53 in glioblastomas correlates with expression of vimentin and glial fibrillary acidic protein. Neurooncology.

[90] Bai L, Zhu W-G. p53: structure, function and therapeutic applications. In: 2006: Journal of Cancer Molecules; 2006: 141–53.

[91] Fu Z, Malureanu L, Huang J, Wang W, Li H, van Deursen JM, Tindall DJ, Chen J (2008). Plk1-dependent phosphorylation of FoxM1 regulates a transcriptional programme required for mitotic progression. Nat Cell Biol.

[92] Sivakumar S, Gorbsky GJ (2015). Spatiotemporal regulation of the anaphase-promoting complex in mitosis. Nat Rev Mol Cell Biol.

[93] Gully CP, Velazquez-Torres G, Shin JH, Fuentes-Mattei E, Wang E, Carlock C, Chen J, Rothenberg D, Adams HP, Choi HH (2012). Aurora B kinase phosphorylates and instigates degradation of p53. Proc Natl Acad Sci USA.

[94] Kan LK, Drummond K, Hunn M, Williams D, O’Brien TJ, Monif M (2020). Potential biomarkers and challenges in glioma diagnosis, therapy and prognosis. BMJ Neurol open.

[95] Hegi ME, Diserens AC, Gorlia T, Hamou MF, de Tribolet N, Weller M, Kros JM, Hainfellner JA, Mason W, Mariani L (2005). MGMT gene silencing and benefit from temozolomide in glioblastoma. N Engl J Med.

[96] Rivera AL, Pelloski CE, Gilbert MR, Colman H, De La Cruz C, Sulman EP, Bekele BN, Aldape KD (2010). MGMT promoter methylation is predictive of response to radiotherapy and prognostic in the absence of adjuvant alkylating chemotherapy for glioblastoma. Neurooncology.

[97] Cheng L, Yuan M, Li S, Lian Z, Chen J, Lin W, Zhang J, Zhong S (2021). Identification of an IFN-beta-associated gene signature for the prediction of overall survival among glioblastoma patients. Annals of Translational Medicine.

[98] Ji H, Ba Y, Ma S, Hou K, Mi S, Gao X, Jin J, Gong Q, Liu T, Wang F (2021). Construction of Interferon-Gamma-Related gene signature to characterize the Immune-Inflamed phenotype of Glioblastoma and Predict Prognosis, Efficacy of Immunotherapy and Radiotherapy. Front Immunol.

[99] Duarte CW, Willey CD, Zhi D, Cui X, Harris JJ, Vaughan LK, Mehta T, McCubrey RO, Khodarev NN, Weichselbaum RR (2012). Expression signature of IFN/STAT1 signaling genes predicts poor survival outcome in glioblastoma multiforme in a subtype-specific manner. PLoS ONE.

[100] Edgar R, Domrachev M, Lash AE (2002). Gene expression Omnibus: NCBI gene expression and hybridization array data repository. Nucleic Acids Res.

