# Supplementary material for: A co-formulation of interferons alpha2b and gamma distinctively targets cell cycle in the glioblastoma-derived cell line U-87MG
Source: BMC Cancer. 2023 Aug 29;23:806. doi: 10.1186/s12885-023-11330-2 (PMC10463508; doi:10.1186/s12885-023-11330-2)
Supplement: Supplementary file 1 — Supplementary Material 1 [file 12885_2023_11330_MOESM1_ESM.docx]

**Additional data file 1:**

**Table S1.** **Oligonucleotides used for qPCR amplifications**. Information of Gene name, GenBank number (GenBank No), Sequence (5’ to 3’) of oligonucleotides in Forward (F) and Reverse (R) directions, number of bases, Product length in PCR and type (Reference, Biomarker or Calibrator) is provided. All were synthesized in Oligonucleotide Synthesis Group (CIGB, Havana). 10^3^ copies of pGEM-T-Easy Vector (Promega, USA) were used as a calibrator in each run.

| **Gene name** | **GenBank No** | **Sequence (5’ to 3’)** | **No Bases** | **Product Length** | **Type** |
| --- | --- | --- | --- | --- | --- |
| GAPDH-F | NM_002046 | TCAACGGATTTGGTCGTATTGG | 22 | 138 | Reference |
| GAPDH-R | NM_002046 | CCATGGGTGGAATCATATTGGA | 22 |  |  |
| HMBS-F | NM_000190 | CCTGACTGGAGGAGTCTGGAGT | 22 | 150 | Reference |
| HMBS-R | NM_000190 | GGAATGTTACGAGCAGTGATGC | 22 |  |  |
| AURKB-F | NM_001284526.1 | CCCATCTGCACTTGTCCTCA | 20 | 144 | Biomarker |
| AURKB-R |  | GGACGCCCAATCTCAAAGTC | 20 |  |  |
| BIRC5-F | NM_001168.2 | CAAGGACCACCGCATCTCTA | 20 | 122 | Biomarker |
| BIRC5-R |  | CCAAGTCTGGCTCGTTCTCA | 20 |  |  |
| BUB1-F | NM_004336.4 | GGTCCGAGGTTAATCCAGCA | 20 | 148 | Biomarker |
| BUB1-R |  | GCCAAAGGAGGAACAACAGG | 20 |  |  |
| BUB1B-F | NM_001211.5 | CCAGGGCCAAAGAGAATGAG | 20 | 125 | Biomarker |
| BUB1B-R |  | TGGAGTGAAACTGGGAAGCA | 20 |  |  |
| CCNB1-F | NM_031966.3 | GAGCCAGAACCTGAGCCTGT | 20 | 78 | Biomarker |
| CCNB1-R |  | TGGGCTTGGAGAGGCAGTAT | 20 |  |  |
| CENPA-F | NM_001809.3 | AGCTCCTGCACCCAGTGTTT | 20 | 118 | Biomarker |
| CENPA-R |  | GCAAAGTCCAGACAGCATCG | 20 |  |  |
| CENPE-F | NM_001813.2 | TGATAGGATGGCGGAGGAAG | 20 | 90 | Biomarker |
| CENPE-R |  | TGGGCAGTTTCTCCAAGTGA | 20 |  |  |
| CENPF-F | NM_016343.3 | TGCCTCTTTGCAGGACACAT | 20 | 138 | Biomarker |
| CENPF-R |  | TGCAGCTCAGTTTCCTTTGC | 20 |  |  |
| FOXM1-F | NM_202002.2 | CAGTGCCAACCGCTACTTGA | 20 | 110 | Biomarker |
| FOXM1-R |  | GCTCTGGATTCGGTCGTTTC | 20 |  |  |
| PLK1-F | NM_005030.4 | TTCGAGGTGGATGTGTGGTC | 20 | 140 | Biomarker |
| PLK1-R |  | GGGTTGATGTGCTTGGGAAT | 20 |  |  |
| ZWINT-F | NM_007057.3 | GAAGACACGAGCCGACAGAA | 20 | 131 | Biomarker |
| ZWINT-R |  | TGGGCTTCCTCCATCTGAGT | 20 |  |  |
| CDC20-F | NM_001255.2 | CTTCCCTGCCAGACCGTATC | 20 | 137 | Biomarker |
| CDC20-R |  | ACCAGAGCTTGCACTCCACA | 20 |  |  |
| pGEM-T-Easy Vector |  | AGCGGATAACAATTTCACACAGGA | 24 |  | Calibrator |
|  |  | CGCCAGGGTTTTCCCAGTCACGAC | 24 |  |  |

**Table S2: Output of Gene Ontology (GO) analysis for 23 common genes.** *Adj p-value<0.05.

| GO Term | Count | % | P-Value | *Benjamini |
| --- | --- | --- | --- | --- |
| defense response to virus | 7 | 36.8 | 2.00E-08 | 4.30E-06 |
| innate immune response | 8 | 42.1 | 2.40E-07 | 2.50E-05 |
| antigen processing and presentation of endogenous peptide antigen via MHC class I via ER pathway, TAP-independent | 3 | 15.8 | 1.80E-05 | 1.30E-03 |
| response to interferon-beta | 3 | 15.8 | 3.50E-05 | 1.90E-03 |
| positive regulation of T cell mediated cytotoxicity | 3 | 15.8 | 2.20E-04 | 9.50E-03 |
| type I interferon signaling pathway | 3 | 15.8 | 6.30E-04 | 2.00E-02 |
| negative regulation of viral genome replication | 3 | 15.8 | 6.60E-04 | 2.00E-02 |
