# Supplementary material for: A co-formulation of interferons alpha2b and gamma distinctively targets cell cycle in the glioblastoma-derived cell line U-87MG
Source: BMC Cancer. 2023 Aug 29;23:806. doi: 10.1186/s12885-023-11330-2 (PMC10463508; doi:10.1186/s12885-023-11330-2)
Supplement: Supplementary file 2 — Supplementary Material 2 [file 12885_2023_11330_MOESM2_ESM.pdf]

Additional data file 2:

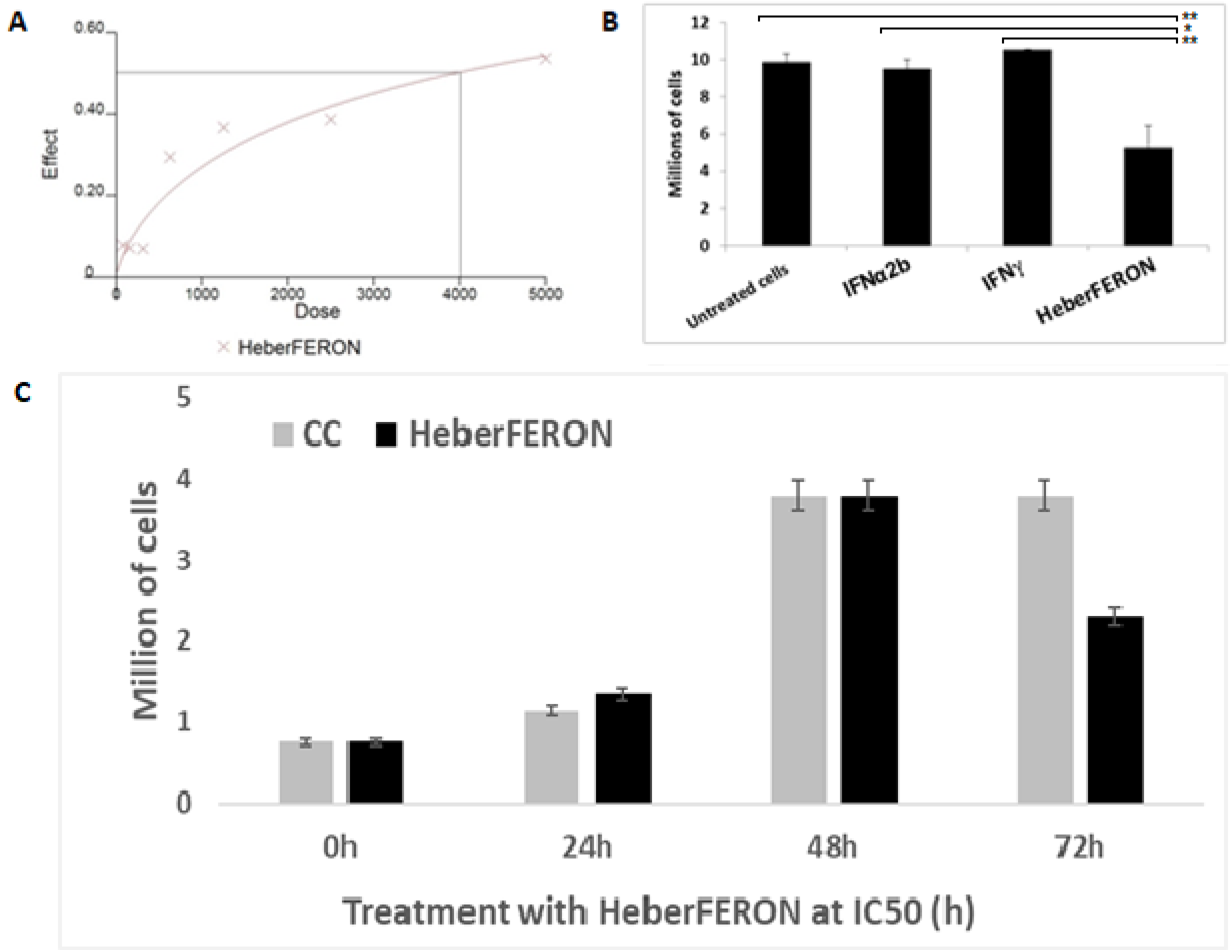

Figure S1. Dose-Effect HeberFERON Curves and cell counts. (**A**) The cells were treated with HeberFERON at different concentrations for 72 h and cell viability was assessed by the MTT assay. The relationship between concentration in IU/mL (Dose) and Effect (0-0.6, meaning 0-60% of cell proliferation inhibition) is plotted. IC50 was calculated as the concentration required to achieve a 50% (0.5) effect. (**B**) Cell counts with Trypan blue (0.4%) was performed in duplicated cultures treated with HeberFERON at IC50 or their equivalent dose for IFNα2b or IFNγ. (**C**) Kinetics of HeberFERON treatment with IC50 dose after 24h, 48h and 72h. The number of cells is given as the average ± SD (standard deviation) in absolute number of cells. The average and standard deviations of millions of cells are shown. The statistically significant differences between

treatments according to an ANOVA with a Tukey's multiple comparisons test are represented as ** p<0.01; * p<0.05.

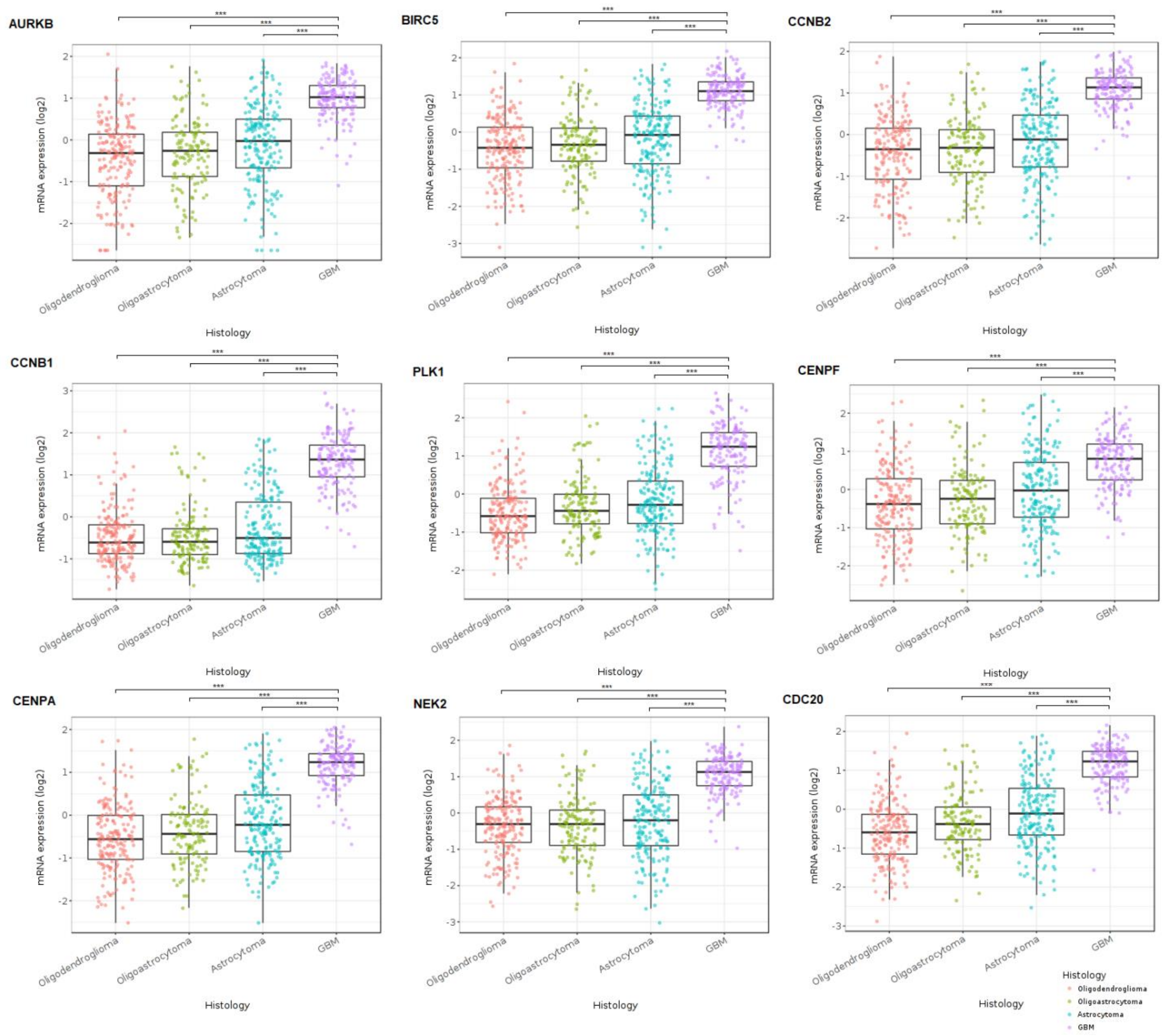

Figure S2: **Expression profiles of FOXM1 regulated genes and CDC20 in brain tumors.** The figure was generated with GlioVis application using data from the TCGA GBMLGG dataset, which include 667 samples from oligodendrogliomas, oligoastrocytomas, astrocytomas and glioblastomas (GBM). Tukey's Honest Significant Difference results of pairwise comparisons between types of brain tumors by histology. The plot shows the difference between pairs and use asterisks according to the level of significance of differences (p-value of the pairwise comparisons), ***p<0.001.

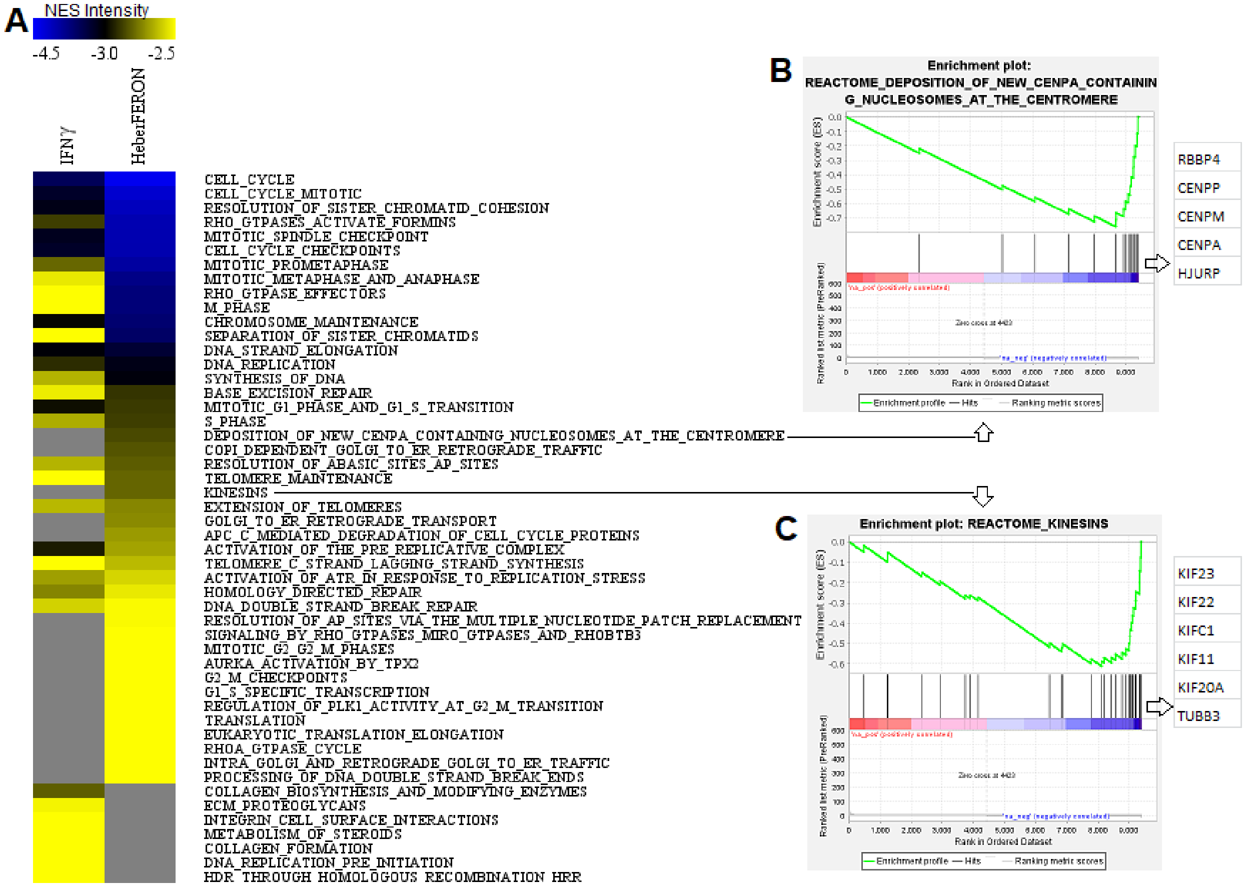

Figure S3: Results of Gene Set Enrichment Analysis (GSEA). A) Heatmap of the NES of Reactome pathways resulting from GSEA analysis. The two columns correspond to IFNγ and HeberFERON. Reactome Pathways ordered by NES for HeberFERON. Identification of signaling distinctively regulated (inhibited) by this combination of IFNs α2b and γ. B/C) Two enrichment plots and the most relevant genes from the Core enrichment set are visualized for Pathways present only under HeberFERON: “Deposition of new CENPA containing nucleosomes at the centromere”(B) and “Kinesins” (C). The most relevant genes for the enrichment significance are listed to the right of each Enrichment Plot.

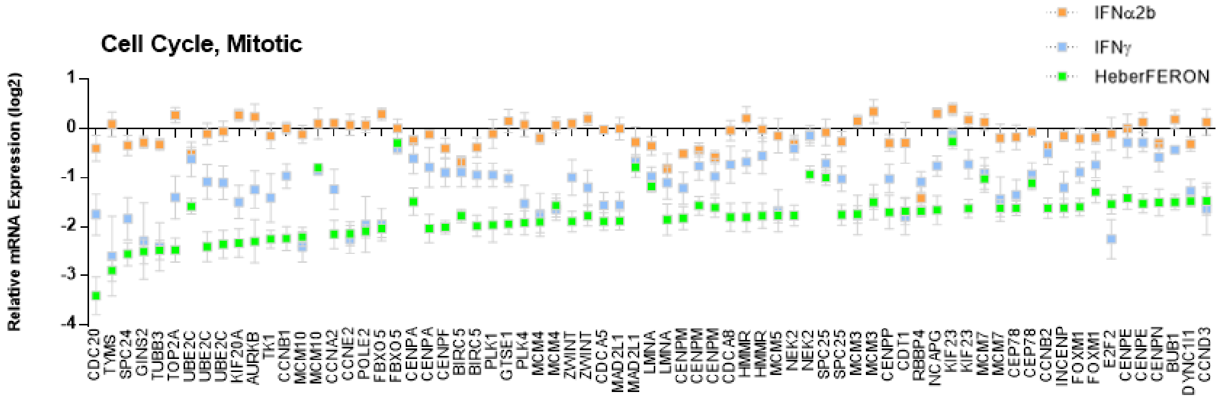

Figure S4: Cell cycle gene expression plot. $\log_2$ expression (mean + SD) relative to averaged controls at probe level are plotted. Orange, blue and green bars correspond to IFNα2b, IFNγ and HeberFERON expression values, respectively. The top 50 genes (71 probes) from the CES of Reactome mitotic cell cycle pathway are shown ordered by the rank metric score.

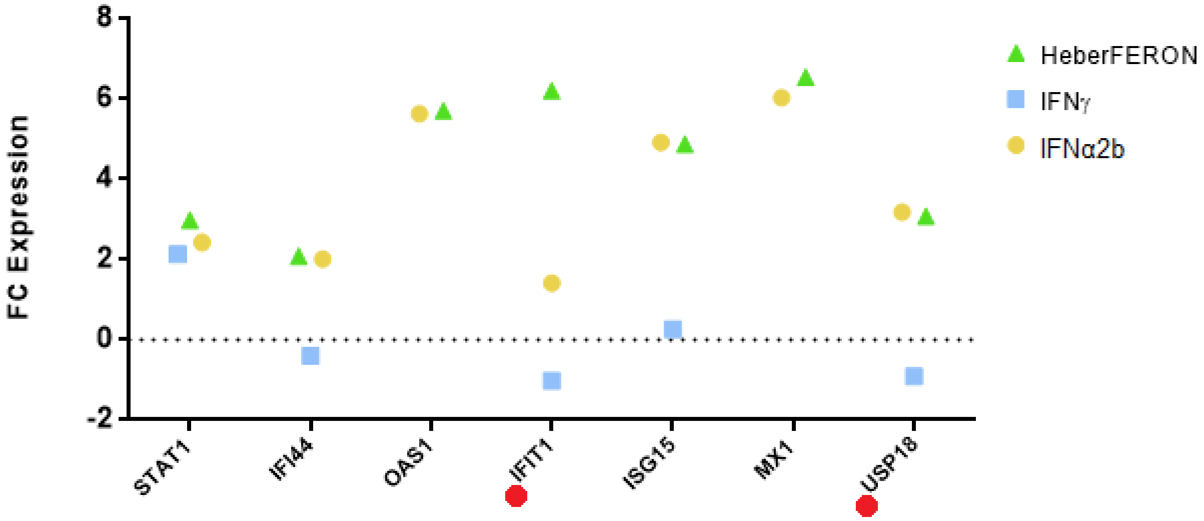

Figure S5: Fold changes of genes from a molecular signature associated to poor prognosis in the Proneural GBM subtype. Orange, blue and green dots represents expression values in samples treated with IFNα2b, IFNγ and HeberFERON, respectively. Red dots highlight genes whose expression is favorable for the disease outcome.
